# Metabarcoding reveals massive species diversity of Diptera in a subtropical ecosystem

**DOI:** 10.1002/ece3.8535

**Published:** 2022-01-23

**Authors:** Junhao Huang, Xiaoqian Miao, Qingyun Wang, Frank Menzel, Pu Tang, Ding Yang, Hong Wu, Alfried P. Vogler

**Affiliations:** ^1^ Department of Forestry Protection School of Forestry and Biotechnology Zhejiang A&F University Hangzhou China; ^2^ Senckenberg Deutsches Entomologisches Institut Müncheberg Germany; ^3^ State Key Laboratory of Rice Biology and Ministry of Agriculture Key Laboratory of Agricultural Entomology Institute of Insect Sciences Zhejiang University Hangzhou China; ^4^ College of Plant Protection China Agricultural University Beijing China; ^5^ Department of Life Sciences Natural History Museum London UK; ^6^ Department of Life Sciences Imperial College London Ascot UK

**Keywords:** COI gene, community structure, high‐throughput sequencing, Malaise trap, two‐winged flies

## Abstract

Diptera is often considered to be the richest insect group due to its great species diversity and broad ecological versatility. However, data on dipteran diversity from subtropical ecosystems have hitherto been scarce, due to the lack of studies conducted at an appropriate large scale. We investigated the diversity and composition of Diptera communities on Tianmu Mountain, Zhejiang, China, using DNA metabarcoding technology, and evaluated their dynamic responses to the effects of slope aspect, season, and altitudinal zone. A total of 5,092 operational taxonomic units (OTUs) were discovered and tentatively assigned to 72 dipteran families, including 2 family records new for China and 30 family records new for the locality. Cecidomyiidae, Sciaridae, and Phoridae were the predominant families, representing 53.6% of total OTUs, while 52 families include >95% unidentified and presumed undescribed species. We found that the community structure of Diptera was significantly affected by aspect, seasonality (month) and elevation, with richer diversity harbored in north‐facing than south‐facing slopes, and seasonality a more profound driver of community structure and diversity than elevation. Overall, massive species diversity of Diptera communities was discovered in this subtropical ecosystem of east China. The huge diversity of potentially undescribed species only revealed by metabarcoding now requires more detailed taxonomic study, as a step toward an evolutionary integration that accumulates information on species’ geographic ranges, ecological traits, functional roles, and species interactions, and thus places the local communities in the context of the growing knowledge base of global biodiversity and its response to environmental change.

## Graphical abstract

Using DNA metabarcoding technology, we investigated the diversity of Diptera communities of a subtropical ecosystem of Tianmu Mountain in eastern China. The result demonstrates the extraordinary species (5092 OTUs) and phylogenetic (72 families) diversity of Diptera, particularly in Cecidomyiidae, Sciaridae, and Phoridae that combined represented 53.6% of total OTUs, while 52 families each contained more than 95% unknown or unidentifiable species, and many more species than recognized at these sites in morphology‐based studies (“excess taxa”). The community structure of Diptera was significantly affected by aspect, seasonality, and, to a lesser extent, elevation.

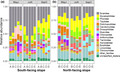

## 1 INTRODUCTION

Insects are the most diverse group of invertebrates on Earth, accounting for more than 50% of all known species in terrestrial and freshwater ecosystems (Schowalter, 2016; Stork, 2018). Although insects constitute only a small proportion of the total biomass, their importance for ecosystem functions and services is profound (Albrecht et al., 2020; Bar‐On et al., 2018; Kunin, 2019; Price et al., 2011; Yang & Gratton, 2014). However, widespread declines in insect diversity and abundance have raised alarm about the future of global biodiversity, after a tremendous biomass reduction was detected across continents during the past few decades, particularly in insect groups that play key roles for various ecosystem processes including pollination, natural pest control, and decomposition (Powney et al., 2019; Seibold et al., 2019; Soroye et al., 2020; Wagner, 2019). However, no net insect abundance and diversity declines were observed in long‐term ecological research sites (Crossley et al., 2020; Outhwaite et al., 2020). Despite the general agreement that Anthropocene forces threaten biodiversity, evidence of wholesale declines of insects remains elusive (Outhwaite et al., 2020; Van Klink et al., 2020). There is an urgent need to develop novel strategies for biodiversity monitoring, which often rely on detailed local inventories for recognizing the species under observation (Creedy et al., 2019; Ji et al., 2020). Thus, in selecting target taxa for inventory, attention should be paid to the groups that play important roles in the structure and function of an ecosystem or geographic area (García‐López et al., 2011).

The Diptera was suggested as an excellent indicator group due to their great diversity of ecological adaptations, life histories, and morphological divergence (Brown et al., 2018; Engels et al., 2020; Yeates et al., 2007). They occur abundantly and display great species diversity with 160,000 named species worldwide, which comprises about 10% of the global described biodiversity (Marshall, 2012; Smith & Mayfield, 2015). Estimates of total Diptera species diversity have been ranging from 400,000 to 800,000 (Pape et al., 2011; Smith & Mayfield, 2015; Srivathsan et al., 2019), but this is likely to be a great underestimate. Recent studies based on DNA barcoding of temperate and tropical ecosystems predicted there are 1.8 million species of Cecidomyiidae alone (Borkent et al., 2018; Hebert et al., 2016). In addition to their high diversity, Diptera have important ecological functions in maintaining terrestrial ecosystems, and great impact on mankind such as human health and agricultural and forestry management, or as models in the study of genetics and forensic science (Skevington & Dang, 2002). All of these roles contribute to the value of Diptera as a suitable indicator of biodiversity. However, compared to beetles and moths which attract many insect enthusiasts, most Diptera are relatively small, dull‐colored, and soft‐bodied species, and thus more difficult to preserve and less attractive to study (Hebert et al., 2016; Morinière et al., 2019). Thus, while representing the potentially largest and taxonomically most challenging insect order, a relatively large proportion of the dipteran species remains unidentifiable and unnamed (Marshall, 2012), in particular in subtropical and tropical areas.

DNA barcoding overcomes some of the limitation of taxonomic identification with classical methods, and it is now widely used in species identification and biomonitoring (Hebert & Cywinska, 2003; Kress & Erickson, 2008; Morinière et al., 2019). Yet, cost limitations of traditional Sanger sequencing or high‐throughput sequencing (HTS) of each individual have severely restricted the possibilities of characterizing millions of specimens required for the study of highly diverse insect groups (Gautier et al., 2013; Polz & Cavanaugh, 1998; Shokralla et al., 2014). Instead, metabarcoding using HTS on the Illumina platform provides a reliable and cost‐effective method for monitoring biodiversity at large scale (Noguerales et al., 2021; Wang et al., 2018). It has proven particularly useful for the study of mixed environmental samples from Malaise traps that capture huge species diversity of Diptera (Arribas et al., 2016; Serrana et al., 2019; Shokralla et al., 2015; Voelkerding et al., 2009). Already, metabarcoding has been widely applied to the study of microbial communities of soil and animal guts, macrozoobenthos in marine and fresh water, and invertebrates from soil and Malaise traps, emerging as a promising way of advancing biodiversity research (Arribas et al., 2016; Elbrecht & Leese, 2015; Ji et al., 2013; Morinière et al., 2019; Orgiazzi et al., 2015; Zhang et al., 2016). It is an efficient method for detecting cryptic species and micro‐insects, which has revealed a huge species diversity of usually neglected species‐rich groups and species only known from sequence data that often are referred to as “dark taxa” (Janzen et al., 2017; Morinière et al., 2019; Page, 2016).

Here, we use metabarcoding to estimate the species diversity and distribution of Diptera in a local biodiversity hotspot, the Tianmu Mountain, located in a subtropical zone of East China at the transition of the Oriental and Palearctic regions (Holt et al., 2013; Wallace, 1958). The flora and insect fauna of this mountain range have been relatively well studied, with 924 species in 51 families of Diptera hitherto recorded (Yang et al., 2016a, 2016b), out of 17,827 species in 113 families known from China (Yang et al., 2020a, 2020b; Yang et al., 2018). Using metabarcoding based on the mitochondrial cytochrome oxidase subunit I (COI) marker, we applied HTS techniques to investigate the diversity of Diptera to answer the following questions: (i) What is the total species diversity and how diverse are local communities in the Tianmu Mountain? (ii) How does the community composition vary with environmental factors, that is, between the different slope aspects and across elevation zones and months of the year (seasonality)? The study will add to a small number of such inventories conducted to date, none of which in the subtropical zone, which will contribute to an improved knowledge of global species diversity of insect and a baseline for studying insect decline.

## 2 MATERIALS AND METHODS

### 2.1 Study sites

Tianmu Mountain is located in Zhejiang Province, East China (119°24′–119°27′E, 30°18′–30°21′N). The site is strongly influenced by typical seasonal monsoon climate and represents a transition from the mid‐subtropics to the northern subtropics (Wang et al., 2017). The annual mean temperature was 15.8°C during the period 2004–2013, with annual precipitation of 1,390–1,870 mm and a relative humidity of 76–81% (Li et al., 2017). This site is favorable for tree growth, and natural forest cover exceeds 95%. The elevation of the highest peak of the site is 1,587 m, with a vertical distribution of the vegetation varying with elevation that consists of evergreen broadleaf forests, mixed evergreen–deciduous broadleaf forests, deciduous broadleaf forests, and shrubs around the peak. The insect fauna of the region is considered to be extremely diverse and community composition to be unique (Wu & Pan, 2001).

### 2.2 Sample collection

Slope aspect (slope orientation facing north or south), elevation, and month periods were selected as environmental factors in this study. Five plots (50 × 50 m) were set on a south‐facing slope and four plots on a north‐facing slope in five elevation zones, representing 320–500 m (zone A), 500–700 m (zone B), 700–850 m (zone C), 850–1,000 m (zone D), and 1,000–1,150 m (zone E). In 2018, samples were collected during three month‐long periods from May to June (abbreviated as MayJ), July to August (JulA), and September to October (SepO). Malaise traps are passive intercept traps for capturing flying insects, which are accepted as an effective measure for general surveys of dipteran diversity (Morinière et al., 2019; Skevington & Dang, 2002). We installed three traps at each plot in the same direction along the slope separated by a distance of about 50 m, with trap head and central axis orientated to the peak. Sites of each trap were of similar canopy density. Specimens were collected and preserved in 100% ethanol. The collection bottles were renewed monthly. A schematic diagram showed the location of the Malaise traps (Figure S1) and collecting information was presented in Table S1. Therefore, there are totally 81 samples in this study, that is (5 elevation zones in south‐facing slope + 4 elevation zones in north‐facing slope) × 3 month periods × 3 Malaise traps (each trap as a biological replicate).

### 2.3 Sample preparation and Illumina sequencing

For each sample, all adult dipterans were selected from the mixed invertebrates. Individuals were sorted into three size classes: <0.5, 0.5‒1.5, and >1.5 cm, referred to as small, medium, and large, respectively (Elbrecht et al., 2017). Samples for bulk DNA extraction were prepared using one leg of large specimens, the thorax of medium‐sized specimens that equal to the tissue of a fly of 0.5 cm, and whole bodies of small specimens (Zhang et al., 2016).

The mixed materials of each sample were dried at room temperature to remove the ethanol and then proteinase K digested overnight at 56°C in lysis buffer. DNA was obtained by a destructive extraction method using the Phenol/Chloroform technique (Green & Sambrook, 2017). PCR was conducted on the DNA pools for 418 bp of the COI barcode region using the primers III_B_F (5′. CCIGAYATRGCITTYCCICG. 3′; Shokralla et al., 2015) and Fol_degen_rev (5′. TANACYTCNGGRTGNCCRAARAAYCA. 3′; Yu et al., 2012). Each sample was amplified for triplicates on an ABI GeneAmp ^®^9700 (Applied Biosystems, USA), following the PCR protocol of Arribas et al. (2016). After adding dual‐index barcodes, purified amplicons were pooled in equimolar proportions (Illumina TruSeqTM DNA Sample Prep Kit). The resulting libraries were paired‐end sequenced (2×300 bp) on the Illumina MiSeq platform (Illumina, San Diego, USA) according to the standard protocols by Majorbio Bio‐Pharm Technology Co., Ltd., Shanghai, China. Sequences of 27 samples of MayJ and 54 samples of JulA and SepO were generated in two runs, respectively.

### 2.4 Sequence data processing

Raw sequencing reads were demultiplexed, quality‐filtered using Trimmomatic v. 0.32 (Bolger et al., 2014) and merged with FLASH v. 1.2.11 (Fast Length Adjustment of Short reads; Magoč & Salzberg, 2011) under the following criteria: (i) The reads were truncated at any site receiving an average quality score <20 over a 50‐bp sliding window, discarding truncated reads shorter than 50 bp and reads containing ambiguous characters; (ii) exact barcode matching and primer mismatch of no more than 2 nucleotide were required; and (iii) sequences whose overlap being longer than 10 bp were merged according to their overlap with mismatch of no more than 2 bp (Table S2). Only contigs with the expected length of 418 bp and unique sequences with ≥5 copies were retained (Creedy et al., 2019). The resulting high‐quality sequences were clustered into operational taxonomic units (OTUs) at 95% (Hebert et al., 2016) similarity cutoff using UPARSE v. 7.1 (http://drive5.com/uparse/) with a greedy algorithm that performs chimera filtering (Edgar, 2013). The taxonomy of each COI gene sequence was analyzed with the RDP Classifier algorithm (http://rdp.cme.msu.edu/) against the NCBI nt database (see Arribas et al., 2016). For the OTUs placed to genus level, the species assignment was further confirmed by sequence similarity in the NCBI database. Those OTUs with >95% similarity to a fully identified entry were assigned with the species name of the given record.

Based on the ratio of observed number of OTUs to the number of individuals of Diptera in each sample, two outliers were replaced by average values (Table S3). After discarding low‐abundance OTUs (total counts <5 reads), the BioConductor package EdgeR was used to normalize the resulting OTUs by the trimmed means of M values method (Robinson et al., 2010). OTU relative abundance in each sample was determined from the result of the normalization and applied in the subsequent analysis.

### 2.5 Data analysis and measures of diversity

In order to gain a deeper understanding of the dipteran diversity of Tianmu Mountain, the proportion of OTU number exceeding the species number recorded from the local (“excess taxa”) and the proportion of OTUs not identified to specific species according to NCBI database (“unidentified taxa”) were calculated. The “unidentified” category includes species that may be known to science, but no relevant sequence data are available to be recognized here, or they may be species not known and only recognized here as distinct sequences, that is, constituting “dark taxa” (Page, 2016). In a few cases, a close hit was recorded to a sequence from an unidentified specimen. Rarefaction curves of the Shannon–Wiener index of each sample reached a plateau, which indicated that the amount of sequencing data is large enough to capture most of the dipteran diversity represented in the samples (Figure S2). The distribution patterns of OTUs among different groups were visualized through Venn diagrams. Diversity indices (Richness, Shannon–Wiener diversity, and Pielou's evenness) of the total communities were calculated using the R package “vegan” (Oksanen et al., 2019; Pielou, 1966; Shannon, 1948). Community composition structure was estimated using Bray–Curtis dissimilarities between samples, and canonical analysis of principal coordinates (CAP) was performed to visualize the differences among samples across seasons and elevation zones (Lozupone & Knight, 2005). Additionally, permutational multivariate analysis of variance (PERMANOVA) was used to test the effect size and significance on the community composition of aspects and month periods (Edwards et al., 2015). Plots and calculations were performed using R v.3.6.0 (R Core Team, 2019). A nonparametric test was performed using SPSS version 20 (Chicago, USA).

## 3 RESULTS

### 3.1 Overall dipteran diversity

**FIGURE 1:**
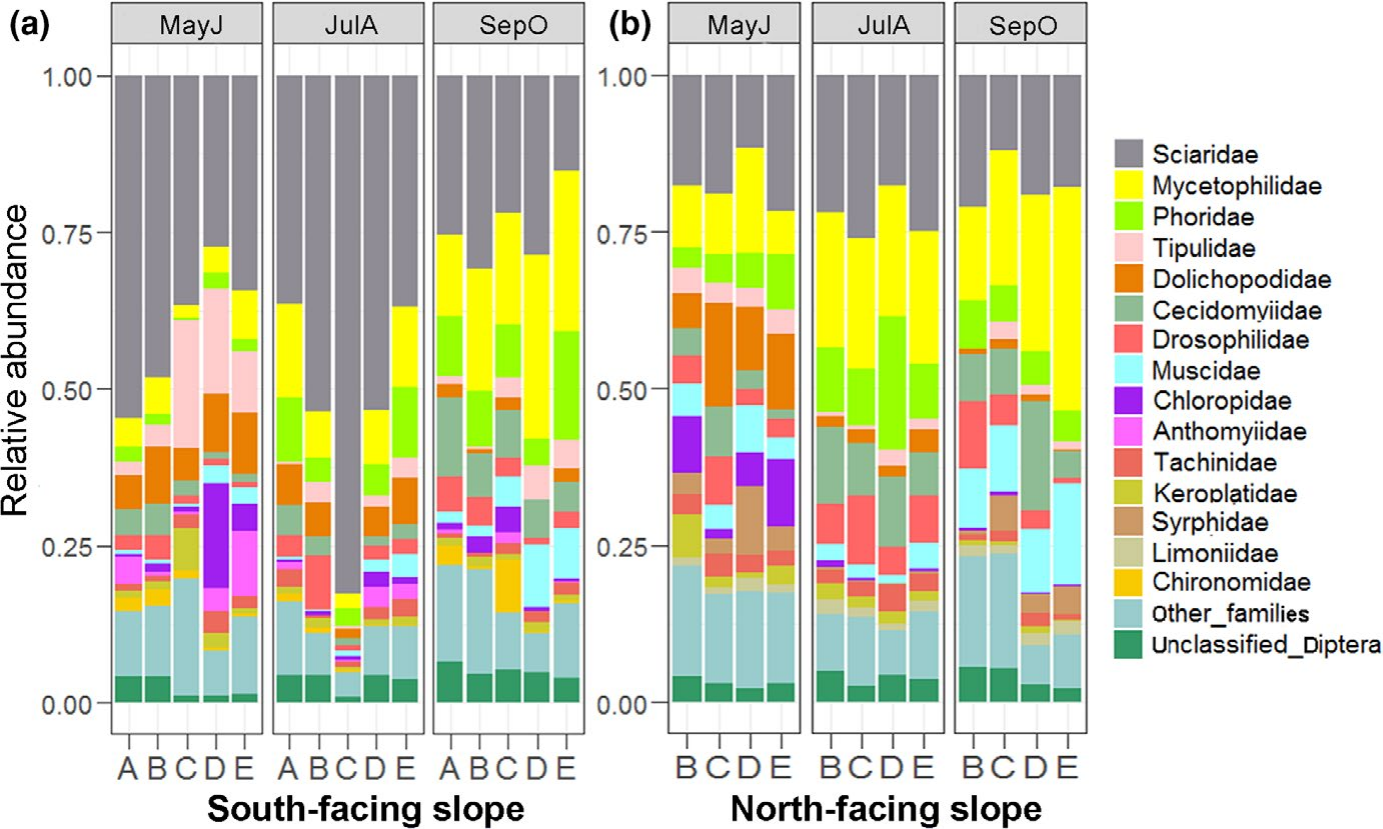
Histograms of dipteran fauna abundance at family level on the south‐facing and north‐facing slopes. Data shown are the top 15 families, and the rest are assigned to the “other_families.” Families are depicted by blocks in different color. Period abbreviations: MayJ, May to June; JulA, July to August; SepO, September to October. Elevation zones: A, 320‒500 m; B, 500‒700 m; C, 700–850 m; D, 850‒1,000 m; E, 1,000‒1,150 m

About 181,000 Diptera individuals in 81 samples were isolated for HTS. In total, 1,489,337 valid sequences were obtained (Table S2), which clustered into 5,092 OTUs after quality filtering. Accepting the identifications from the RDP classifier, OTUs were classified into 72 families, covering five nematoceran infraorders (Ptychopteromorpha, Tipulomorpha, Psychodomorpha, Culicomorpha, Bibionomorpha) and the suborder Brachycera. Bibionomorpha (57%, 45%) and Brachycera (30%, 48%) were dominant on both the south‐facing and north‐facing slopes (Figure S3). The Brachycera was represented by 50 families, which accounted for 69% of the community at family level (Table S4). Sciaridae (39%, 19%), Mycetophilidae (12%, 19%), and Phoridae (6%, 8%) were the dominant families of both aspects (Figure 1 and Figure S4).

At the level of OTUs, Cecidomyiidae proved to be extraordinarily species rich, comprising 1,529 OTUs and accounting for 30% of the total diversity, followed by Sciaridae (698 OTUs, 14%) and Phoridae (500 OTUs, 10%) (Table S4). Thirty‐seven families contained more than 80% of the OTUs, accounting for 51% of all families (Table S4). The ratios of unknown taxa and median body size were negatively correlated: The smaller the average body size of a family, the higher both the ratio of "excess taxa" and "unidentified taxa" (Figure S5). Also remarkable was the detection of two families new to China (Rangomaramidae and Aulacigastridae) and thirty families new to Tianmu Mountain. In addition, 151 OTUs could not be identified to the family level (Table S4).

Moreover, 2,205 OTUs could be identified to genus level. Among them, five dominant genera comprised 56.9% of the total dipteran read abundance, including *Bradysia* (Sciaridae, 28.2%), *Megaselia* (Phoridae, 11.3%), *Drosophila* (Drosophilidae, 6.5%), *Phaonia* (Muscidae, 5.9%), and *Mycomya* (Mycetophilidae, 5.1%). Species assignment of these OTUs was further confirmed by the identity of sequences against the NCBI database, which resulted in 173 OTUs that finally were assigned to specific species (Table S5) and additional 49 OTUs found perfect matches in the database but their taxonomic assignment only was to genus level. Nearly half of these 173 species identified OTUs were from Drosophilidae (41 OTUs), Sciaridae (21 OTUs), and Tachinidae (17 OTUs).

### 3.2 Endemic and shared taxa

**FIGURE 2:**
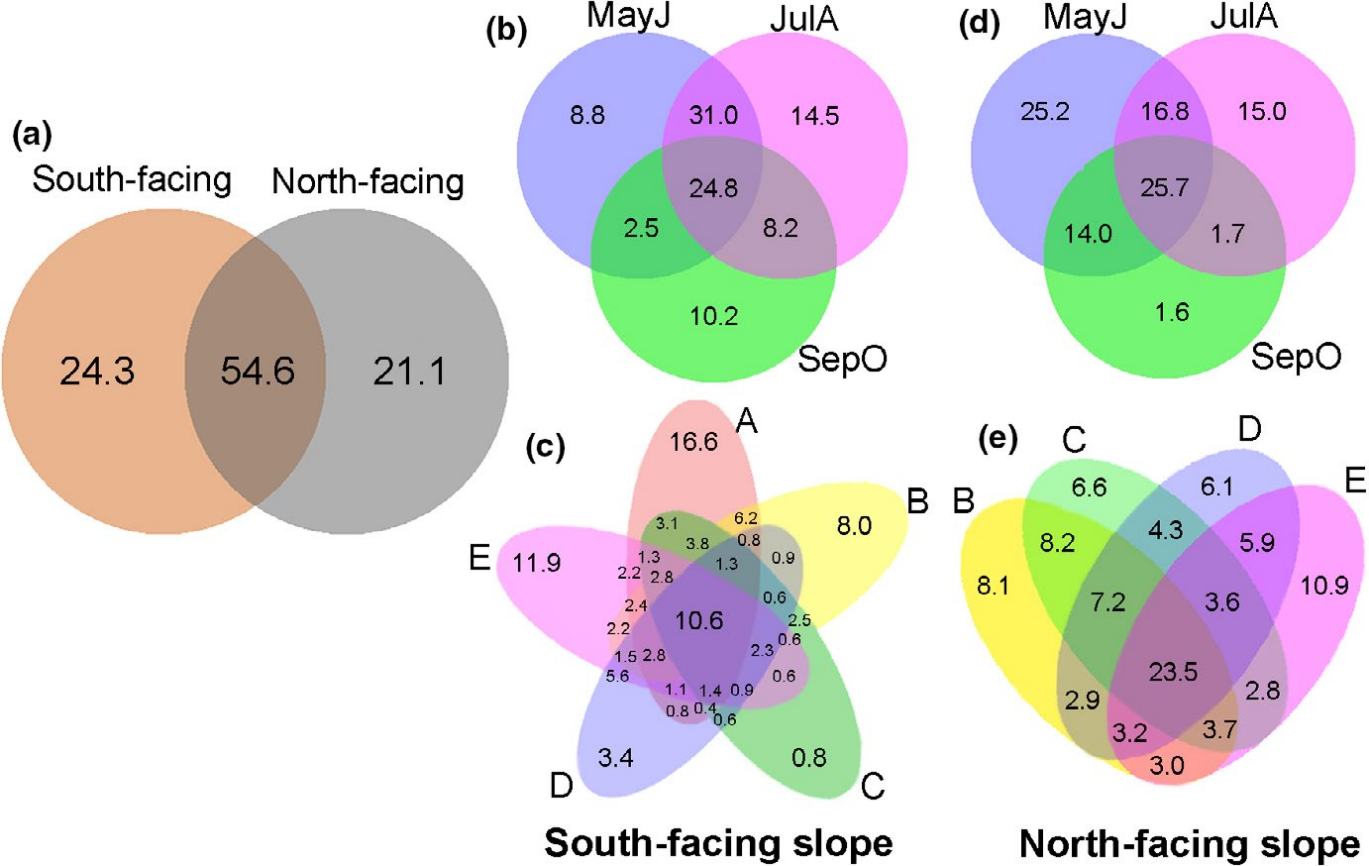
Venn diagram showing exclusive and shared OTUs of Diptera. (a) Total fauna on south‐facing and north‐facing slopes. (b, c) Month periods and elevations on the south‐facing slope. (d, e) Month periods and elevations on the north‐facing slope. Period abbreviations: MayJ, May to June; JulA, July to August; SepO, September to October. Elevation zones: A, 320‒500 m; B, 500‒700 m; C, 700–850 m; D, 850‒1,000 m; E, 1,000‒1,150 m

There were noteworthy overlaps in widespread OTUs among different treatments, with 54.6% OTUs shared between south‐facing and north‐facing slopes (Figure 2). With an additional site at the lowest elevation (320–500 m), more OTUs were discovered on the south‐facing slopes (5 sites), including 4,017 OTUs in 66 families, compared to 3,857 OTUs in 68 families on north‐facing slopes (4 sites). A total of 1,235 OTUs and four families were exclusive to the south‐facing slope, while 1,075 OTUs and six families were exclusive to the north‐facing slope (Figure 2a). Most OTUs sampled in May to June (MayJ) and July to August (JulA) showed noticeable overlap (62.0% on the south‐facing slope; 43.1% on the north‐facing slope; Figure 2b). All of the families were detected in these periods, except for Rangomaramidae, which was exclusive to September to October (SepO) (Figure 2b,d). Most OTUs were shared in the five elevation zones, and the endemic OTUs were richest in elevation zone A (320–500 m) and E (1,000–1,150 m) on both aspects (Figure 2c,e).

### 3.3 Diversity indices

**FIGURE 3:**
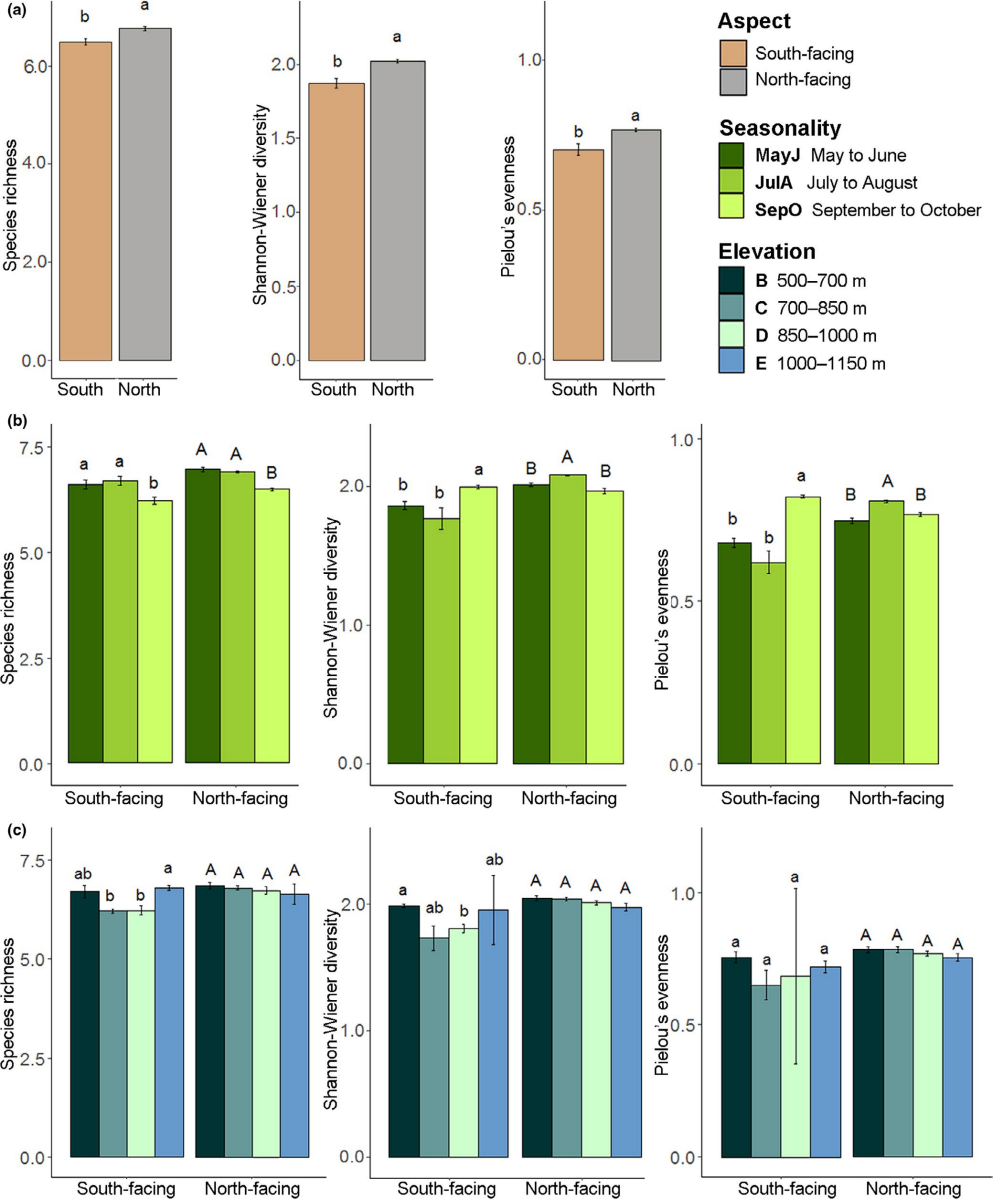
Effects of slope aspect, seasonality, and elevation on species richness, Shannon–Wiener diversity, and Pielou's evenness of Diptera. (a) Effect of aspect. (b) Effect of seasonality. (c) Effect of elevation. Different letters indicate significant differences in a Kruskal–Wallis test at *p* < .05

The community structure of flies in Tianmu Mountain differed significantly between the slope aspects, seasons, and elevation zones. For each site, diversity indices including species richness (alpha‐diversity), Shannon–Wiener diversity, and Pielou's evenness were significantly higher than on the north‐facing than the south‐facing slope (Figure 3a). For both aspects, richness in MayJ and JulA was significantly higher than SepO (Figure 3b). Diversity and evenness of JulA were significantly higher than other periods on the north‐facing slope, while those of SepO were highest on the south‐facing slope (Figure 3b). However, the indices showed no significant differences across the four elevation zones (500–700 m, 700–850 m, 850–1,000 m, and 1,000–1,150 m) on the north‐facing slope. On the south‐facing slope, diversity of elevation zone B (500–700 m) was significantly higher than elevation zone D (850–1,000 m) and richness of elevation zone E (1,000–1,150 m) differed significantly from other elevation zones (Figure 3b).

### 3.4 Community composition

**FIGURE 4:**
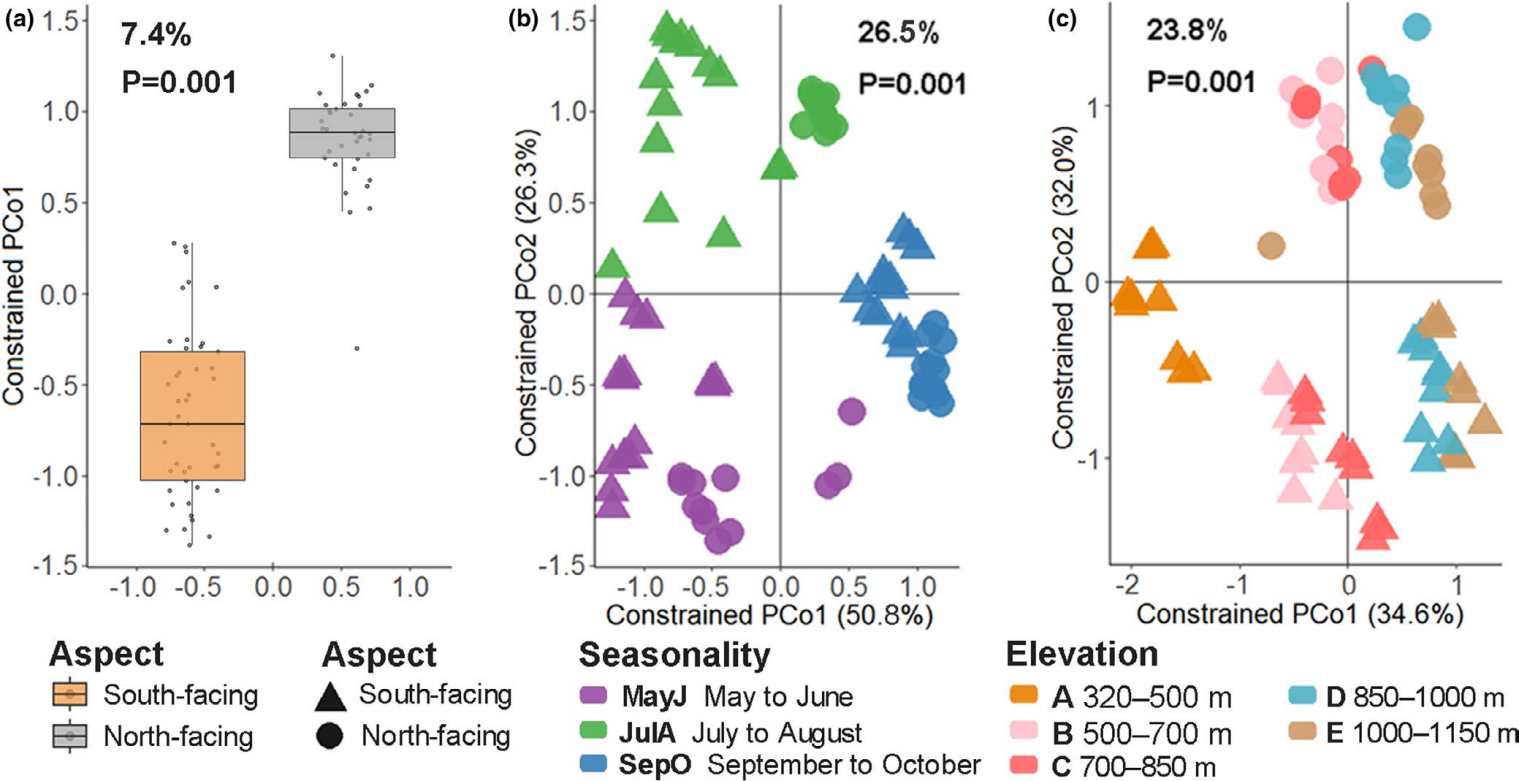
Constrained effects of slope aspect, seasonality, and elevation on dipteran diversity, by partial canonical analysis of principal coordinates using the Bray–Curtis distances. (a) Constrained to the aspects and conditioned by month periods and elevations; (b) constrained to the aspects and month periods, while conditioned by elevations; (c) constrained to the aspects and elevations, while conditioned by month periods

Both CAP and PERMANOVA analysis showed the dipteran community to be affected by aspect, which explained 7.4% and 8.4% of the variation, respectively (*p* = .001) (Table 1 and Table S6, Figure 4a). The cluster dendrogram notably separated all samples into three month‐long periods for both aspects (Figure S6). Month (season) accounted for 28.8% and 33.5% of explained variance by CAP on the south‐facing and north‐facing slopes, respectively (Figure S6a,c), while in the PERMANOVA month accounted for 21.5% of community variation (*p* = .001) (Table 1). CAP analysis also suggested that elevation accounted for 32.8% and 19.4% of explained variance, respectively, for both aspects (Figure S6b,d), while PERMANOVA results showed that elevation explained 18% of the variation in the community (*p* = .001) (Table 1).

**TABLE 1:** Effects of slope aspect, month, elevation, and their interaction on dipteran fauna by PERMANOVA

Effect	*df*	SumsOfSqs	MeanSqs	F. Model	*R* ^2^	*p*
Aspect	1	2.072	2.072	57.779	0.084	.001
Month	2	5.311	2.655	74.038	0.215	.001
Elevation	4	4.498	1.124	31.349	0.182	.001
Aspect × Month	2	2.096	1.048	29.214	0.085	.001
Aspect × Elevation	3	1.907	0.636	17.723	0.077	.001
Month × Elevation	8	4.222	0.528	14.715	0.171	.001
Aspect × Month × Elevation	6	2.610	0.435	12.125	0.106	.001
Residuals	54	1.937	0.036		0.079	
Total	80	24.651			1	

Abbreviations: *df*, the degree of freedom (statistics); *F*. Model, *F*‐test value; MeanSqs, quadratic mean deviation; *p* < .05, significant difference between groups; *R* ^2^, the ratio of the variance of the group to the total variance that indicates the degree of interpretation of sample differences in different groups; SumsOfSqs, total variance.

The results of CAP and PERMANOVA also indicated significant interactive effects of aspect/month (26.5% and 8.5%) and aspect/elevation (23.8% and 7.7%) on the variation within the dipteran community (Figure 4b, c, Table 1). Additionally, the interactive effect of aspect/month/elevation explained 10.6% of the dipteran community variation (Table 1).

## 4 DISCUSSION

### 4.1 Massive diversity of Diptera in subtropical ecosystem

Metabarcoding revealed an unexpectedly high level of dipteran species diversity in Tianmu Mountain. A total of 5,092 OTUs representing 72 families were detected based on HTS, equivalent to 54% of the dipteran families and 29% of the species recorded in China to date (Yang et al., 2020a, 2020b), and exceeding the diversity previously recorded in Tianmu Mountain by 138% at the family level and 552% at the species level (Yang et al., 2016a, 2016b). The findings indicate that existing dipteran species diversity surveys have been inadequate in spite of the great efforts invested in the former morphological studies (Wu & Pan, 2001; Yang et al., 2016a, 2016b). Traditional morphological taxonomic methods are not well suited for generating complete inventories of minute dipterans, while metabarcoding makes it easier to portray the complete diversity in complex Malaise samples.

However, as most OTUs could not be identified morphologically, the OTU or Barcode Index Number (BIN) (Ratnasingham & Hebert, 2007) assignments remain to be validated. OTUs in this study were clustered at the 95% similarity level, following barcoding data of Canadian insects that Diptera had mean nearest‐neighbor divergences averaging 5.44 ± 0.26% (mean ± *SD*), with the Cecidomyiidae (5.06 ± 0.04%) and Sciaridae (4.82 ± 0.07%) showing no evidence of unusually low sequence divergences (Hebert et al., 2016). Consequently, the 1,529 and 698 OTUs placed into Cecidomyiidae and Sciaridae are considered a good approximation of the actual species present in the sample. In fact, these threshold values are conservative, given that most metabarcoding studies of invertebrate diversity have applied a 97% similarity threshold, for example, when characterizing arthropod communities from the tropical forest canopy (Creedy et al., 2019; Zhang et al., 2016), the soil mesofauna (Arribas et al., 2016), and stream macroinvertebrate biodiversity (Serrana et al., 2019). In addition, individual barcoding and large‐scale integrative taxonomy of Sweden scuttle flies (Phoridae) suggested a threshold of 3% for most species of this species rich family (Hartop et al., 2021; Meier et al., 2006).

The total of 5,092 OTUs at Tianmu Mountain is roughly the same number as the 5,092 OTUs of Diptera gathered over a 7‐year period as part of a Malaise trap program across habitat types in Germany, and a corresponding metabarcoding study across the Bavarian Forest that recovered 1,735 OTUs (Morinière et al., 2019). In Great Britain, one of the world's best studied dipteran faunas, about 7,000 dipteran species have been recorded in total (Brown et al., 2018). These comparisons provide a context for the high diversity of Diptera in the subtropical Tianmu Mountain, a single mountain site and one year's investigation. Recent biodiversity research also revealed an astounding species diversity of Diptera in the tropics, while a large‐scale DNA barcoding study of Canadian insects from Malaise traps also showed a huge BIN count of Diptera (Brown et al., 2018; Hebert et al., 2016; Srivathsan et al., 2019). Based on these kinds of studies using samples from passive traps and metabarcoding, it is now widely accepted that the actual species diversity in Diptera in tropical and temperate regions has been greatly underestimated and the Diptera may constitute the most species‐rich insect order, exceeding the Coleoptera (Borkent et al., 2018). Currently, there are 35,153 species in 139 families of Coleoptera recorded in China (Nie et al., 2019), surpassing the known dipteran species diversity of the nation, but with the increasing popularity of metabarcoding, the true species diversity of Diptera may be shown to greatly exceed that of the Coleoptera. Our study also demonstrated the almost complete absence of representation of the Tianmu Mountain assemblage in the existing barcode databases. *Braydysia* was the most species diverse and most abundant genus of the family in Tianmu Mountain, but no matching sequence was found for its most abundant species, which accounted for 17.5% of total dipteran abundance by sequence read numbers. Among all 5,092 dipteran OTUs discovered in Tianmu Mountain, only 173 OTUs (comprising 15.4% of total abundance) were identified to the specific species according to the public databases. In 52 families over 95% of OTU clusters had no database entry, including the three most diverse families, that is, Cecidomyiidae (no match to 99.7% of sequences; 1,529 OTUs), Sciaridae (97.0%; 698 OTUs), and Phoridae (99.4%; 500 OTUs). Among them, Cecidomyiidae accounted for 30% of the dipteran species diversity in Tianmu Mountain. These least studied families include much more species than currently described, with nearly 99% hitherto unrecorded species, which is consistent with the findings of the Canadian barcoding efforts that also detected huge numbers of previously unaccounted species in these same families (Hebert et al., 2016; Marshall, 2012; Yeates et al., 2009). In part, this is explained by their small body sizes, which tends to delay species description (Stork et al., 2015) and is correlated with a greater proportion of unknown taxa and a greater "excess" over the number of known species recoded from Tianmu previously (Figure S5; also see Morinière et al., 2019).

While metabarcoding provides a fast, reliable, and cost‐effective method for approximating species diversity and for monitoring community structures without specific taxonomic expertise (Arribas et al., 2016; Bush et al., 2017; Zhang et al., 2016), the resolution of the identification using this approach depends on the available reference databases and method of classification. Using the RDP classifier, OTUs could generally be placed at the family level, but this is fraught with uncertainties, given that existing morphospecies libraries with linked barcode sequences usable for taxonomic assignment have large gaps, in particular in tropical species (Magdalena et al., 2016; Sinniger et al., 2016), and a total of 151 OTUs in our study could not be assigned even to family level. Methods of sequence‐based classification are notoriously sensitive to the algorithm and the representation in databases, and these assignments therefore can only be used as a preliminary taxonomic placement.

Only the comprehensive analysis of these groups from metabarcoding of many more sites, combined with phylogenetic studies of the major lineages and their species diversity, will refine our evolutionary hypotheses about what causes the richness at Tianmu Mountain, and in particular the apparently unrivaled species diversity in Cecidomyiidae, Sciaridae, and Phoridae. All three families are known as typical dominant groups in forest ecosystems (Borkent et al., 2018; Brown, 2005; Morinière et al., 2019; Ševčík et al., 2016). Together with their extremely high diversity, Cecidomyiidae and Phoridae show various feeding traits, such as phytophagy, parasitoidism, saprophagy, fungivory, and predatory lifestyle, and occur in complex habitats including organic detritus, living plants, and even nests of social insects (Gagne & Moser, 2013; Marshall, 2012; Tokuda, 2012). Sciaridae are mainly phytosaprophagous or mycetophagous and are abundantly present in moist habitats (Menzel & Mohrig, 2000; Miao et al., 2020; Yang et al., 2019). Cecidomyiidae show a peculiarity in developmental and reproduction biology that various species exhibit chromosome elimination, a form of hap‐lo‐di‐ploidy (Kloc & Zagrodzinska, 2010; Tokuda, 2012), while some mycetophagous species of the family have shortened metamorphosis and develop directly by paedogenesis during the larval stage (Hodin & Riddiford, 2001; Marshall, 2012). Linking the ecological and taxonomic diversity in these lineages will require more detailed knowledge about the major clades within the families, their feeding style and habitat associations, biogeographic distributions, and their time of origin and evolutionary change through geological time.

As phylogenetic analyses of Diptera are being refined (e.g., Wiegman et al., 2011), barcode sequences can be placed into the phylogenetic trees with greater precision. Metabarcoding can be combined with shotgun sequencing of mixed community samples for assembly of mitochondrial genomes for well‐supported phylogenetic trees even in otherwise unknown species. This has been shown for the Diptera in a Bornean rainforest that also demonstrated the overwhelming diversity of Sciaridae and Cecidomyiidae, and small‐bodied lineages generally, while providing the phylogenetic framework for placement of the local assemblage relative to samples from elsewhere (Choo et al., 2017). Short metabarcoding sequences can be placed in a mitogenome tree with some confidence, in particular as these trees are increasingly densely sampled. Detailed phylogenetic trees are needed to address critical questions about, for example, the biogeographic origin of lineages or their rate of radiation. The Tianmu Mountain study site at the boundary of the Oriental and Palaearctic zoogeographic regions also will allow the use of phylogenetic methods to establish the affinities to mostly tropical vs. mostly temperate lineages and their respective species diversity. This type of analysis goes beyond the placement conducted with k‐mer‐based classifiers used here, which produces potentially over‐confident placement, and in any case does not go beyond the family level for most OTUs, that is, has limited resolution of important life history or biogeographic characters that vary greatly *within* these diverse families.

### 4.2 Higher species richness and diversity in the north‐facing slope

Prior to the availability of a phylogenetic tree, the Tianmu species diversity can be studied for overall trends in OTU numbers and spatial and temporal turnover at the local site. Total species richness (gamma diversity) at a site or region will be affected by the proportion of turnover. Our study suggests that turnover of Diptera communities is strongly structured by three abiotic factors, aspect, season, and elevation. Significantly higher species richness and diversity per sampling plot were observed on the north‐facing compared to the south‐facing slope, presumably related to variation in sun exposure that influence environmental variables differently by altering the light, temperature, humidity, and food resources of the habitat (Bartlett et al., 2020; Giersch et al., 2017; Jeffries et al., 2006; Salgado et al., 2020; Weiss et al., 2013). In this study area of subtropical secondary broadleaf forest, the south‐facing slope receives more solar radiation and has closed canopy cover with a moist forest floor, while the north‐facing slope is often shaded, with a relatively open canopy and dry forest floor. Seasonal oscillations, particularly temperature and precipitation, further contribute to environmental differentiation, with significant effects on arthropod populations by affecting reproduction, development, and food webs (Kizito et al., 2017; Mohammed & Chadee, 2011; Tonkin et al., 2017; Tun‐Lin et al., 2010; Wilke et al., 2016).

Finally, the altered mesoclimate along the altitudinal gradient is another driving force of insect community composition (Hodkinson, 2005; Hopkins et al., 2018; Mani, 1962; Mckie et al., 2005). The activity periods of Diptera and their diversity have been found to be greatly affected by elevation, such as the delayed appearance of the fruit fly, *Rhagoletis cerasi*, correlated with increased elevation in Turkey (Kovanci & Kovanci, 2006), and the diversity increase of Empidinae dance flies (Empididae) along elevation gradients in the tropics (Chatelain et al., 2018). However, our results suggest that the community variation of Diptera was affected more significantly by seasonality (month periods) than elevation (Table 1, Figure S7).

## 5 CONCLUSION

Metabarcoding approaches can reveal trends of turnover and correlations with spatial, environmental, and phenological parameters, on the scale of entire communities, to tease apart the most important ecological determinants of alpha and beta diversity in unstudied areas. Subsequent analyses based on evolutionary placements of OTUs will refine these conclusions in regard to lineage‐specific responses. This study of a subtropical ecosystem of Tianmu Mountain in eastern China demonstrates the extraordinary species and phylogenetic (family level) diversity of Diptera, particularly in the families Cecidomyiidae, Sciaridae, and Phoridae that combined represented 53.6% of total OTUs, while 52 families each contained more than 95% unknown or unidentifiable species, and many more species than recognized at these sites in morphology‐based studies ("excess taxa"). The community structure of Diptera was significantly affected by aspect, seasonality, and, to a lesser extent, elevation. As the metabarcoding technology matures, an increasingly comprehensive ecological and evolutioary assessment of even the most diverse insect groups can be attempted. A key challenge is the integration of data from different sites, ecosystems, and biogeographic regions that links the information from individual site‐based studies for a global perspective on insect biodiversity.

## CONFLICT OF INTEREST

The authors declare that they have no conflict of interest for the publication of this study.

## AUTHOR CONTRIBUTION

**Junhao Huang:** Conceptualization (lead); Data curation (supporting); Formal analysis (supporting); Funding acquisition (lead); Investigation (supporting); Methodology (equal); Project administration (lead); Resources (lead); Supervision (lead); Validation (lead); Visualization (equal); Writing – original draft (equal); Writing – review & editing (lead). **Xiaoqian Miao:** Data curation (equal); Formal analysis (lead); Investigation (lead); Visualization (equal); Writing – original draft (equal). **Qingyun Wang:** Data curation (supporting); Methodology (supporting); Project administration (supporting); Writing – original draft (equal); Writing – review & editing (supporting). **Frank Menzel:** Writing – review & editing (equal). **Pu Tang:** Conceptualization (equal); Investigation (supporting); Project administration (supporting); Writing – review & editing (equal). **Ding Yang:** Project administration (equal); Supervision (equal); Writing – review & editing (equal). **Hong Wu:** Project administration (equal); Supervision (equal); Writing – review & editing (equal). **Alfried P. Vogler:** Conceptualization (equal); Methodology (equal); Project administration (equal); Supervision (equal); Writing – review & editing (lead).

## Supporting information

Supplementary Material

## Data Availability

The sequencing data supporting the results of this study are available in Dryad Digital Repository (https://doi.org/10.5061/dryad.8kprr4xq2).
