# Supplementary material for: Metabarcoding reveals massive species diversity of Diptera in a subtropical ecosystem
Source: Ecol Evol. 2022 Jan 23;12(1):e8535. doi: 10.1002/ece3.8535 (PMC8796913; doi:10.1002/ece3.8535)
Supplement: Supplementary file 1 — Supplementary Material [file ECE3-12-e8535-s001.docx]

**Appendix**

**Table S1.** Collection information of the insect samples in this study. Month abbreviations: MayJ, May to June; JulA, July to August; SepO, September to October.

| **Aspect** | **Elevation** | **Coordinate** | **Period**  **abbreviation** | **Sampling month period** | **Collector** |
| --- | --- | --- | --- | --- | --- |
| **South-facing slope** | A: 320–500 m | 30.316314 N  119.443995 E | MayJ  JulA  SepO | 2018.05–2018.06  2018.07–2018.08  2018.09–2018.10 | Xiaoqian Miao |
|  | B: 500–700 m | 30.331327 N  119.437434 | MayJ  JulA  SepO | 2018.05–2018.06  2018.07–2018.08  2018.09–2018.10 | Xiaoqian Miao |
|  | C: 700–850 m | 30.333183 N  119.433233 E | MayJ  JulA  SepO | 2018.05–2018.06  2018.07–2018.08  2018.09–2018.10 | Xiaoqian Miao |
|  | D: 850–1,000 m | 30.333317 N  119.417009 E | MayJ  JulA  SepO | 2018.05–2018.06  2018.07–2018.08  2018.09–2018.10 | Xiaoqian Miao |
|  | E: 1,000–1,150 m | 30.342125 N  119.434841 E | MayE  JulE  SepE | 2018.05–2018.06  2018.07–2018.08  2018.09–2018.10 | Xiaoqian Miao |
| **North-facing slope** | B: 500–700 m | 30.400516 N  119.416350 E | MayJ  JulA  SepO | 2018.05–2018.06  2018.07–2018.08  2018.09–2018.10 | Xiaoqian Miao |
|  | C: 700–850 m | 30.383783 N  119.400717 E | MayJ  JulA  SepO | 2018.05–2018.06  2018.07–2018.08  2018.09—2018.10 | Xiaoqian Miao |
|  | D: 850–1,000 m | 30.394245 N  119.411449 E | MayJ  JulA  SepO | 2018.05–2018.06  2018.07–2018.08  2018.09–2018.10 | Xiaoqian Miao |
|  | E: 1,000–1,150 m | 30.386528 N  119.411169 E | MayJ  JulA  SepO | 2018.05–2018.06  2018.07–2018.08  2018.09–2018.10 | Xiaoqian Miao |

**Table S2.** Number of sequences in different steps of data processing.

| Raw reads | High-quality  sequences | Valid sequences | Sequences with  OTUs abundance≥5 | Sequences after normalization |
| --- | --- | --- | --- | --- |
| 3,288,250 | 1,583,080 | 1,520,466 | 1,489,337 | 1,493,797 |

**Table S3.** The information of quality control in dipteran sequence analysis for each sample.

| **Sample**  **ID** | **Dipteran**  **individual**  **number**  **in three traps** | **OTUs** | | | **Ratio of OTU number / individual number** | | |  |
| --- | --- | --- | --- | --- | --- | --- | --- | --- |
|  |  | **Trap 1** | **Trap 2** | **Trap 3** | **Trap 1** | **Trap 2** | **Trap 3** |  |
| South-facing slope | | | | | | | | |
| TMayJA | 7,621 | 747 | 751 | 712 | 9.8% | 9.9% | 9.3% | |
| TMayJB | 5,975 | 948 | 928 | 983 | 15.9% | 15.5% | 16.5% | |
| TMayJC | 2,179 | 457 | 467 | 497 | 21.0% | 21.4% | 22.8% | |
| TMayJD | 4,291 | 525 | 585 | 593 | 12.2% | 13.6% | 13.8% | |
| TMayJE | 5,291 | 727 | 671 | 742 | 13.7% | 12.7% | 14.0% | |
| TJulAA | 4,142 | 633 | 666 | 910 | 15.3% | 16.1% | 22.0% | |
| TJulAB | 4,522 | 602 | 690 | 819 | 13.3% | 15.3% | 18.1% | |
| TJulAC | 8,786 | 431 | 440 | 645 | 4.9% | 5.0% | 7.3% | |
| TJulAD | 7,007 | 606 | 623 | 884 | 8.6% | 8.9% | 12.6% | |
| TJulAE | 9,453 | 786 | 812 | 945 | 8.3% | 8.6% | 10.0% | |
| TSepOA | 884 | 673 | 764 | 748 | 76.1% | 86.4% | 84.6% | |
| TSepOB | 696 | 330 | 337 | 333 | 47.4% | 48.4% | 47.8% | |
| TSepOC | 1,959 | 535 | 549 | 568 | 27.3% | 28.0% | 29.0% | |
| TSepOD | 752 | 337 | 350 | 342 | 44.8% | 46.5% | 45.5% | |
| TSepOE | 1,697 | 490 | 496 | 497 | 28.9% | 29.2% | 29.3% | |
| North-facing slope | | | | | | | | |
| LMayJB | 10,848 | 1252 | 1275 | 1339 | 11.5% | 11.8% | 12.3% |  |
| LMayJC | 9,616 | 995 | 1020 | 1057 | 10.3% | 10.6% | 11.0% |  |
| LMayJD | 9,280 | 926 | 949 | 1093 | 10.0% | 10.2% | 11.8% |  |
| LMayJE | 9,752 | 759 | 888 | 1139 | 7.8% | 9.1% | 11.7% |  |
| LJulAB | 5,699 | 947 | 957 | 1017 | 16.6% | 16.8% | 17.8% |  |
| LJulAC | 6,292 | 1028 | 960 | 1055 | 16.3% | 15.3% | 16.8% |  |
| LJulAD | 7,984 | 1072 | 1080 | 1056 | 13.4% | 13.5% | 13.2% |  |
| LJulAE | 5,762 | 938 | 907 | 912 | 16.3% | 15.7% | 15.8% |  |
| LSepOB | 1,398 | 710 | 735 | 707 | 50.8% | 52.6% | 50.6% |  |
| LSepOC | 1,465 | 703 | 822 | 734 | 48.0% | 56.1% | 50.1% |  |
| LSepOD | 717 | 601 | 566 | 600 | 83.8% | 78.9% | 83.7% |  |
| LSepOE | 1,857 | 544 | 603 | 564 | 29.3% | 32.5% | 30.4% |  |

**Table S4.** Dipteran OTUs number of each family in this study, compared to reported species numbers from Tianmu Mountain, China and the World.

| **Higher taxa** | **Family** | **OTUs**  **number** | **Species**  **Identified**  **OTUs** | **Species**  **Reported**  **in Tianmu** | **Species**  **reported**  **in China** | **Species**  **of world** | **% of**  **excess**  **taxa** | **% of**  **unidentified**  **taxa** | **Body size**  **(mm)** |
| --- | --- | --- | --- | --- | --- | --- | --- | --- | --- |
| Ptychopteromorpha | Ptychopteridae | 3 | 0 | 1 | 15 | 75 | 66.67 | 100.00 | 7.0–15.0 |
| Tipulomorpha | Cylindrotomidae | 1 | 0 | 0 | 19 | – | 100.00 | 100.00 | 11.0–16.0 |
| Tipulomorpha | Limoniidae | 47 | 1 | 23 | 691 | – | 51.06 | 97.87 | 2.0–11.0 |
| Tipulomorpha | Pediciidae | 3 | 0 | 0 | 34 | 500 | 100.00 | 100.00 | 5.0–35.0 |
| Tipulomorpha | Tipulidae | 63 | 3 | 33 | 553 | 15,457 | 47.62 | 95.24 | 7.0–35.0 |
| Psychodomorpha | Anisopodidae | 2 | 0 | 0 | 5 | 159 | 100.00 | 100.00 | 4.0–12.0 |
| Psychodomorpha | Psychodidae | 49 | 3 | 0 | 80 | 2,958 | 100.00 | 93.88 | 2.0–6.0 |
| Psychodomorpha | Trichoceridae | 4 | 0 | 0 | 14 | 160 | 100.00 | 100.00 | 3.0–9.0 |
| Culicomorpha | Ceratopogonidae | 143 | 0 | 31 | 1,176 | 6,267 | 78.32 | 100.00 | 1.0–5.0 |
| Culicomorpha | Chironomidae | 238 | 14 | 75 | 828 | 7,054 | 68.49 | 94.12 | 1.0–10.0 |
| Culicomorpha | Culicidae | 61 | 13 | 12 | 419 | 3,550 | 80.33 | 78.69 | 3.0–9.0 |
| Culicomorpha | Dixidae | 3 | 0 | 0 | 14 | 186 | 100.00 | 100.00 | 3.0–5.5 |
| Culicomorpha | Simuliidae | 13 | 2 | 0 | 333 | 2,132 | 100.00 | 84.62 | 1.2–6.0 |
| Bibionomorpha | Bibionidae | 5 | 0 | 8 | 126 | 760 | N/A | 100.00 | 2.0–15.0 |
| Bibionomorpha | Cecidomyiidae | 1,529 | 5 | 8 | 180 | 6,203 | 99.48 | 99.67 | 0.5–3.0 |
| Bibionomorpha | Diadocidiidae | 2 | 1 | 0 | 4 | 34 | 100.00 | 50.00 | 3.0–5.0 |
| Bibionomorpha | Keroplatidae | 46 | 0 | 0 | 39 | 945 | 100.00 | 100.00 | 4.0–15.0 |
| Bibionomorpha | Lygistorrhinidae | 1 | 0 | 0 | 3 | 33 | 100.00 | 100.00 | 1.0–6.0 |
| Bibionomorpha | Mycetophilidae | 334 | 3 | 20 | 263 | 4,164 | 94.01 | 99.10 | 2.0–15.0 |
| Bibionomorpha | Rangomaramidae | 1 | 0 | 0 | 0 | – | 100.00 | 100.00 | 1.7–6.0 |
| Bibionomorpha | Scatopsidae | 2 | 0 | 0 | 9 | 390 | 100.00 | 100.00 | 3.0–12.0 |
| Bibionomorpha | Sciaridae | 698 | 21 | 11 | 400 | 2,800 | 98.42 | 96.99 | 1.0–6.0 |
| Brachycera | Agromyzidae | 46 | 0 | 0 | 169 | 2,977 | 100 | 100.00 | 1.0–6.0 |
| Brachycera | Anthomyiidae | 65 | 3 | 19 | 671 | 1,927 | 70.77 | 95.38 | 4.0–12.0 |
| Brachycera | Asilidae | 28 | 1 | 10 | 305 | 7,479 | 64.29 | 96.43 | 8.0–20.0 |
| Brachycera | Aulacigastridae | 1 | 0 | 0 | 0 | – | 100 | 100.00 | 2.0–5.0 |
| Brachycera | Calliphoridae | 22 | 5 | 16 | 313 | 1,522 | 27.27 | 77.27 | 4.0–16.0 |
| Brachycera | Canacidae | 2 | 0 | 0 | 10 | – | 100 | 100.00 | 1.6–5.0 |
| Brachycera | Carnidae | 2 | 0 | 0 | 1 | – | 100 | 100.00 | 1.0–2.5 |
| Brachycera | Celyphidae | 2 | 0 | 3 | 45 | – | N/A | 100.00 | 3.0–5.0 |
| Brachycera | Chloropidae | 49 | 0 | 8 | 315 | 2,880 | 83.67 | 100.00 | 1.0–5.0 |
| Brachycera | Clusiidae | 2 | 0 | 0 | 6 | 360 | 100 | 100.00 | 1.5–8.0 |
| Brachycera | Conopidae | 3 | 0 | 7 | 91 | 783 | N/A | 100.00 | 5.0–15.0 |
| Brachycera | Ctenostylidae | 1 | 1 | 1 | 5 | 10 | 0 | 0.00 | 4.0–9.0 |
| Brachycera | Curtonotidae | 6 | 0 | 0 | 1 | 80 | 100 | 100.00 | – |
| Brachycera | Diopsidae | 4 | 0 | 1 | 30 | – | 75 | 100.00 | 4.0–10.0 |
| Brachycera | Dolichopodidae | 145 | 1 | 42 | 1096 | 7,236 | 71.03 | 99.31 | 1.0–9.0 |
| Brachycera | Drosophilidae | 209 | 41 | 0 | 953 | 4,315 | 100 | 80.38 | 1.5–7.0 |
| Brachycera | Dryomyzidae | 3 | 1 | 0 | 3 | – | 100 | 66.67 | 5.0–18.0 |
| Brachycera | Empididae | 100 | 1 | 27 | 552 | 3,142 | 73 | 99.00 | 1.0–12.0 |
| Brachycera | Ephydridae | 15 | 1 | 3 | 191 | 1,992 | 80 | 93.33 | 1.0–11.0 |
| Brachycera | Fanniidae | 7 | 0 | 5 | 135 | 359 | 28.57 | 100.00 | 2.0–5.0 |
| Brachycera | Heleomyzidae | 12 | 0 | 0 | 18 | 728 | 100 | 100.00 | 1.2–12.0 |
| Brachycera | Lauxaniidae | 28 | 2 | 15 | 300 | 1,895 | 46.43 | 92.86 | 2.0–7.0 |
| Brachycera | Lonchaeidae | 4 | 0 | 0 | 24 | 504 | 100 | 100.00 | 3.0–6.0 |
| Brachycera | Lonchopteridae | 4 | 0 | 1 | 26 | – | 75 | 100.00 | 2.0–5.0 |
| Brachycera | Micropezidae | 1 | 0 | 0 | 29 | 579 | 100 | 100.00 | 3.0–16.0 |
| Brachycera | Milichiidae | 9 | 0 | 0 | 39 | 364 | 100 | 100.00 | 1.0–6.0 |
| Brachycera | Muscidae | 87 | 7 | 47 | 1710 | 5,210 | 45.98 | 91.95 | 2.0–18.0 |
| Brachycera | Opomyzidae | 2 | 0 | 1 | 4 | – | 50 | 100.00 | 2.0–5.0 |
| Brachycera | Pallopteridae | 2 | 0 | 0 | 3 | – | 100 | 100.00 | 2.5–7.0 |
| Brachycera | Periscelididae | 1 | 0 | 0 | 3 | 127 | 100 | 100.00 | 1.0–5.0 |
| Brachycera | Phoridae | 500 | 3 | 19 | 226 | 4,105 | 96.2 | 99.40 | 0.5–6.0 |
| Brachycera | Pipunculidae | 9 | 1 | 2 | 85 | 1,420 | 77.78 | 88.89 | 2.0–12.0 |
| Brachycera | Platypezidae | 1 | 0 | 0 | 8 | – | 100 | 100.00 | 1.5–6.0 |
| Brachycera | Platystomatidae | 7 | 0 | 2 | 71 | – | 71.43 | 100.00 | 3.0–11.0 |
| Brachycera | Psilidae | 10 | 0 | 3 | 67 | – | 70 | 100.00 | 2.5–10.0 |
| Brachycera | Rhagionidae | 5 | 0 | 5 | 85 | 711 | 0 | 100.00 | 2.0–20.0 |
| Brachycera | Rhiniidae | 1 | 0 | 10 | 71 | 190 | N/A | 100.00 | 5.0–12.0 |
| Brachycera | Rhinophoridae | 2 | 0 | 0 | 1 | – | 100 | 100.00 | 2.0–11.0 |
| Brachycera | Sarcophagidae | 10 | 3 | 35 | 332 | 3,094 | N/A | 70.00 | 3.0–22.0 |
| Brachycera | Scathophagidae | 6 | 1 | 0 | 40 | 414 | 100 | 83.33 | 3.0–12.0 |
| Brachycera | Sciomyzidae | 3 | 0 | 1 | 56 | – | 66.67 | 100.00 | 2.0–14.0 |
| Brachycera | Sepsidae | 5 | 0 | 6 | 59 | 384 | N/A | 100.00 | 2.0–6.0 |
| Brachycera | Sphaeroceridae | 63 | 3 | 25 | 164 | 1,880 | 60.32 | 95.24 | 0.7–5.5 |
| Brachycera | Stratiomyidae | 13 | 1 | 25 | 346 | 2,666 | N/A | 92.31 | 2.0–25.0 |
| Brachycera | Syrphidae | 59 | 11 | 68 | 956 | 6,016 | N/A | 81.36 | 3.5–35.0 |
| Brachycera | Tabanidae | 9 | 0 | 27 | 459 | 4,406 | N/A | 100.00 | 6.0–30.0 |
| Brachycera | Tachinidae | 116 | 17 | 202 | 1253 | 8,500 | N/A | 85.34 | 2.0–20.0 |
| Brachycera | Tephritidae | 10 | 2 | 32 | 623 | 4,911 | N/A | 80.00 | 2.5–10.0 |
| Brachycera | Therevidae | 1 | 0 | 2 | 36 | – | N/A | 100.00 | 2.5–15.0 |
| Brachycera | Xylophagidae | 1 | 1 | 0 | 305 | 136/138 | 100 | 0.00 | 5.0–11.0 |
| Unclassified_Diptera |  | 151 |  |  |  |  |  |  |  |

Note: “–” indicates unclear data; “% of excess taxa” indicates ratios of OTUs in each family that is over the recorded species number from Tianmu Mountain; “N/A” represents OTUs number less than species reported in Tianmu Mountain; “% of unidentified taxa” indicates ratios of OTUs in each family that not identified to the specific species according to the public barcoding databases. Number of species reported in Tianmu Mountain from Yang *et al.* (2016a, b); number of species reported in China from Yang *et al.* (2018; 2020a, b); number of world species mostly from Pape & Thompson (2013) and Borkent *et al.* (2018).

**Table S5.** The 173 OTUs assigned to specific species.

| **OTUs** | **Higher taxa** | **Family** | **Species** | **New to**  **China** | **Relative**  **abundance** | **Percent**  **identity** | **Genbank**  **acssesion no.** |
| --- | --- | --- | --- | --- | --- | --- | --- |
| OTU6873 | Tipulomorpha | Limoniidae | *Dicranomyia frontalis* | yes | 0.001% | 96.65% | KR971750.1 |
| OTU3965 | Tipulomorpha | Tipulidae | *Angarotipula illustris* | yes | 0.010% | 95.44% | HM416279.1 |
| OTU3349 | Tipulomorpha | Tipulidae | *Pselliophora guangxiensis* |  | 0.002% | 97.85% | MF095105.1 |
| OTU3571 | Tipulomorpha | Tipulidae | *Tipula biprocessa* |  | 0.010% | 97.61% | KY861850.1 |
| OTU2555 | Psychodomorpha | Psychodidae | *Clogmia albipunctata* |  | 0.028% | 100.00% | MK234696.1 |
| OTU1240 | Psychodomorpha | Psychodidae | *Psychoda uncinula* |  | 0.003% | 98.09% | MT745811.1 |
| OTU5439 | Psychodomorpha | Psychodidae | *Trichadenotecnum subrotundus* |  | 0.026% | 100.00% | MT408531.1 |
| OTU4759 | Culicomorpha | Chironomidae | *Chironomus flaviplumus* |  | 0.015% | 95.45% | JF412075.1 |
| OTU1870 | Culicomorpha | Chironomidae | *Chironomus fujitertius* | yes | 0.007% | 97.85% | LC494822.1 |
| OTU1484 | Culicomorpha | Chironomidae | *Chironomus javanus* |  | 0.005% | 100.00% | KT212985.1 |
| OTU3133 | Culicomorpha | Chironomidae | *Chironomus striatipennis* | yes | 0.004% | 99.52% | LC495119.1 |
| OTU3740 | Culicomorpha | Chironomidae | *Phaenopsectra flavipes* |  | 0.001% | 95.69% | KC250831.1 |
| OTU1640 | Culicomorpha | Chironomidae | *Polypedilum asakawaense* | yes | 0.003% | 100.00% | LC329175.1 |
| OTU3061 | Culicomorpha | Chironomidae | *Polypedilum benokiense* |  | 0.007% | 96.89% | MG949865.1 |
| OTU4191 | Culicomorpha | Chironomidae | *Polypedilum cultellatum* | yes | 0.001% | 97.37% | MG949886.1 |
| OTU6328 | Culicomorpha | Chironomidae | *Polypedilum henicurum* | yes | 0.216% | 100.00% | MG950086.1 |
| OTU6052 | Culicomorpha | Chironomidae | *Polypedilum japonicum* | yes | 0.052% | 98.33% | MG950031.1 |
| OTU4961 | Culicomorpha | Chironomidae | *Polypedilum tsukubaense* | yes | 0.001% | 98.80% | MG950083.1 |
| OTU1929 | Culicomorpha | Chironomidae | *Rheocricotopus villiculus* |  | 0.016% | 100.00% | MT456593.1 |
| OTU226 | Culicomorpha | Chironomidae | *Smittia aterrima* |  | 0.008% | 97.37% | LC462333.1 |
| OTU2861 | Culicomorpha | Chironomidae | *Tanytarsus shoudigitatus* |  | 0.022% | 98.80% | KT613548.1 |
| OTU2707 | Culicomorpha | Culicidae | *Aedes albopictus* | yes | 0.040% | 99.76% | KY765465.1 |
| OTU1947 | Culicomorpha | Culicidae | *Aedes flavopictus* |  | 0.008% | 98.80% | NC_050044.1 |
| OTU4766 | Culicomorpha | Culicidae | *Aedes japonicus* | yes | 0.001% | 98.09% | KM457597.1 |
| OTU6570 | Culicomorpha | Culicidae | *Aedes nipponicus* | yes | 0.002% | 98.56% | KT358406.1 |
| OTU2014 | Culicomorpha | Culicidae | *Aedes watasei* | yes | 0.005% | 97.13% | AB738120.1 |
| OTU2509 | Culicomorpha | Culicidae | *Anopheles bengalensis* | yes | 0.011% | 95.70% | GQ259190.1 |
| OTU4047 | Culicomorpha | Culicidae | *Culex kyotoensis* |  | 0.014% | 99.04% | LC104327.1 |
| OTU5910 | Culicomorpha | Culicidae | *Culex pseudovishnui* |  | 0.006% | 99.52% | AB738094.1 |
| OTU6359 | Culicomorpha | Culicidae | *Culex tritaeniorhynchus* |  | 0.017% | 100.00% | KX574844.1 |
| OTU6568 | Culicomorpha | Culicidae | *Culex tritaeniorhynchus* |  | 0.010% | 99.52% | MW488913.1 |
| OTU5591 | Culicomorpha | Culicidae | *Culex vagans* |  | 0.007% | 99.76% | JQ306320.1 |
| OTU2677 | Culicomorpha | Culicidae | *Uranotaenia novobscura* |  | 0.006% | 99.28% | LC054531.1 |
| OTU3646 | Culicomorpha | Simuliidae | *Simulium chomthongense* |  | 0.008% | 98.55% | KF289464.1 |
| OTU3904 | Culicomorpha | Simuliidae | *Simulium muangpanense* |  | 0.003% | 98.56% | LC509569.1 |
| OTU2702 | Bibionomorpha | Cecidomyiidae | *Asphondylia tojoi* | yes | 0.007% | 98.09% | LC373199.2 |
| OTU583 | Bibionomorpha | Cecidomyiidae | *Asphondylia yushimai* |  | 0.033% | 100.00% | LC348703.1 |
| OTU235 | Bibionomorpha | Cecidomyiidae | *Feltiella acarisuga* |  | 0.003% | 100.00% | AB698982.1 |
| OTU3760 | Bibionomorpha | Cecidomyiidae | *Lasioptera yoichiensis* | yes | 0.004% | 95.44% | MN509490.1 |
| OTU1622 | Bibionomorpha | Cecidomyiidae | *Thecodiplosis japonensis* |  | 0.001% | 95.45% | AB105484.1 |
| OTU1825 | Bibionomorpha | Diadocidiidae | *Diadocidia cizeki* |  | 0.665% | 95.74% | KJ136804.1 |
| OTU3666 | Bibionomorpha | Mycetophilidae | *Exechia separata* | yes | 0.001% | 96.17% | KM679372.1 |
| OTU3489 | Bibionomorpha | Mycetophilidae | *Leia winthemii* | yes | 0.002% | 99.04% | KY830183.1 |
| OTU4039 | Bibionomorpha | Mycetophilidae | *Sciophila pseudoflexuosa* | yes | 0.012% | 98.56% | KM679394.1 |
| OTU784 | Bibionomorpha | Sciaridae | *Bradysia atracornea* | yes | 0.009% | 98.56% | JX418069.1 |
| OTU6380 | Bibionomorpha | Sciaridae | *Bradysia cuneiforma* | yes | 0.101% | 95.67% | JX418051.1 |
| OTU4384 | Bibionomorpha | Sciaridae | *Bradysia fugaca* | yes | 0.007% | 96.17% | JX418103.1 |
| OTU2333 | Bibionomorpha | Sciaridae | *Bradysia hilariformis* | yes | 0.005% | 96.89% | KU923071.1 |
| OTU267 | Bibionomorpha | Sciaridae | *Bradysia procera* | yes | 0.029% | 99.76% | JX418153.1 |
| OTU2151 | Bibionomorpha | Sciaridae | *Cratyna ambigua* | yes | 0.005% | 98.80% | JQ613829.1 |
| OTU5294 | Bibionomorpha | Sciaridae | *Dolichosciara semiferruginea* |  | 0.009% | 95.45% | KX981167.1 |
| OTU3184 | Bibionomorpha | Sciaridae | *Dolichosciara semiferruginea* |  | 0.220% | 98.56% | KX981178.1 |
| OTU5944 | Bibionomorpha | Sciaridae | *Phytosciara ussuriensis* | yes | 0.177% | 96.65% | JQ613848.1 |
| OTU5036 | Bibionomorpha | Sciaridae | *Pseudoaerumnosa tkoci* | yes | 0.003% | 95.23% | MW680300.1 |
| OTU5678 | Bibionomorpha | Sciaridae | *Pseudoaerumnosa tkoci* | yes | 0.145% | 100.00% | MW680298.1 |
| OTU4397 | Bibionomorpha | Sciaridae | *Pseudolycoriella microcteniuni* |  | 0.079% | 99.52% | MW239657.1 |
| OTU907 | Bibionomorpha | Sciaridae | *Scatopsciara necopinata* |  | 0.020% | 95.69% | JQ613821.1 |
| OTU5120 | Bibionomorpha | Sciaridae | *Schwenckfeldina custodiata* |  | 0.038% | 99.52% | JQ613849.1 |
| OTU1035 | Bibionomorpha | Sciaridae | *Sciara humeralis* |  | 0.001% | 97.22% | JF870998.1 |
| OTU4645 | Bibionomorpha | Sciaridae | *Sciara humeralis* |  | 0.020% | 97.85% | KR661089.1 |
| OTU1245 | Bibionomorpha | Sciaridae | *Sciara ruficauda* |  | 1.664% | 99.76% | NC_046767.1 |
| OTU961 | Bibionomorpha | Sciaridae | *Sciara ruficauda* |  | 0.054% | 98.80% | JQ613808.1 |
| OTU6792 | Bibionomorpha | Sciaridae | *Sciara thoracica* | yes | 0.001% | 99.76% | KX950764.1 |
| OTU301 | Bibionomorpha | Sciaridae | *Zygoneura sciarina* |  | 0.154% | 96.17% | JQ613809.1 |
| OTU6816 | Brachycera | Anthomyiidae | *Melanomya nana* | yes | 0.210% | 97.13% | LC500560.1 |
| OTU6175 | Brachycera | Anthomyiidae | *Pegomya circumpolaris* | yes | 0.279% | 95.22% | KT959568.1 |
| OTU6419 | Brachycera | Anthomyiidae | *Pollenia huangshanensis* |  | 0.095% | 98.33% | MN688573.1 |
| OTU856 | Brachycera | Asilidae | *Orophotus mandarinus* |  | 0.127% | 97.37% | MG967826.1 |
| OTU6831 | Brachycera | Calliphoridae | *Aldrichina grahami* |  | 0.005% | 100.00% | MN537823.1 |
| OTU6411 | Brachycera | Calliphoridae | *Chrysomya pinguis* |  | 0.006% | 100.00% | KY020777.1 |
| OTU6398 | Brachycera | Calliphoridae | *Lucilia porphyrina* |  | 0.073% | 100.00% | MF694301.1 |
| OTU6266 | Brachycera | Calliphoridae | *Melinda pusilla* |  | 0.005% | 99.28% | MG968016.1 |
| OTU1361 | Brachycera | Calliphoridae | *Polleniopsis mongolica* |  | 0.166% | 97.60% | MN131052.1 |
| OTU3870 | Brachycera | Ctenostylidae | *Sinolochmostylia sinica* | yes | 0.002% | 98.33% | KR262673.1 |
| OTU4378 | Brachycera | Dolichopodidae | *Dolichopus bigeniculatus* | yes | 0.008% | 95.70% | KT225294.1 |
| OTU4490 | Brachycera | Drosophilidae | *Amiota furcata* | yes | 0.008% | 95.93% | KX034670.1 |
| OTU1613 | Brachycera | Drosophilidae | *Amiota onchopyga* | yes | 0.011% | 100.00% | MG844356.1 |
| OTU2037 | Brachycera | Drosophilidae | *Collessia kirishimana* | yes | 0.012% | 100.00% | LC194124.1 |
| OTU4617 | Brachycera | Drosophilidae | *Drosophila barutani* | yes | 0.016% | 99.28% | DQ471586.1 |
| OTU6212 | Brachycera | Drosophilidae | *Drosophila busckii* | yes | 0.005% | 95.45% | HQ982232.1 |
| OTU4010 | Brachycera | Drosophilidae | *Drosophila busckii* | yes | 0.008% | 99.76% | HQ982461.1 |
| OTU1823 | Brachycera | Drosophilidae | *Drosophila fengkainensis* | yes | 0.018% | 100.00% | AB669754.1 |
| OTU6408 | Brachycera | Drosophilidae | *Drosophila immigrans* | yes | 0.665% | 100.00% | AB824803.1 |
| OTU4653 | Brachycera | Drosophilidae | *Drosophila lacertosa* | yes | 0.072% | 100.00% | AY750095.1 |
| OTU1055 | Brachycera | Drosophilidae | *Drosophila lacertosa* | yes | 0.007% | 96.85% | DQ471590.1 |
| OTU6733 | Brachycera | Drosophilidae | *Drosophila lacertosa* | yes | 0.003% | 95.71% | DQ471590.1 |
| OTU6326 | Brachycera | Drosophilidae | *Drosophila lucipennis* | yes | 0.036% | 99.52% | KJ500328.1 |
| OTU3486 | Brachycera | Drosophilidae | *Drosophila melanogaster* | yes | 0.008% | 100.00% | MT474915.1 |
| OTU80 | Brachycera | Drosophilidae | *Drosophila suzukii* | yes | 0.365% | 100.00% | MG816026.1 |
| OTU2644 | Brachycera | Drosophilidae | *Drosophila tani* | yes | 1.697% | 99.76% | AB669709.1 |
| OTU1418 | Brachycera | Drosophilidae | *Drosophila trapezifrons* | yes | 0.179% | 99.04% | AB825958.1 |
| OTU6438 | Brachycera | Drosophilidae | *Drosophila triauraria* | yes | 0.016% | 100.00% | AB669711.1 |
| OTU4964 | Brachycera | Drosophilidae | *Drosophila trilutea* | yes | 1.459% | 99.52% | LC057190.1 |
| OTU6282 | Brachycera | Drosophilidae | *Hirtodrosophila fascipennis* | yes | 0.005% | 100.00% | LC194064.1 |
| OTU1802 | Brachycera | Drosophilidae | *Leucophenga cultella* | yes | 0.011% | 99.76% | KX069285.1 |
| OTU1911 | Brachycera | Drosophilidae | *Leucophenga longipenis* | yes | 0.013% | 99.76% | KU565765.1 |
| OTU6297 | Brachycera | Drosophilidae | *Leucophenga maculata* | yes | 0.123% | 100.00% | KP697191.1 |
| OTU4246 | Brachycera | Drosophilidae | *Leucophenga neointerrupta* | yes | 0.008% | 100.00% | KC861398.1 |
| OTU1916 | Brachycera | Drosophilidae | *Leucophenga nigrinervis* | yes | 0.019% | 99.52% | KF026566.1 |
| OTU2254 | Brachycera | Drosophilidae | *Leucophenga pentapunctata* |  | 0.050% | 100.00% | KF026585.1 |
| OTU4661 | Brachycera | Drosophilidae | *Leucophenga quadripunctata* |  | 0.002% | 99.04% | KC861400.1 |
| OTU2971 | Brachycera | Drosophilidae | *Leucophenga shillongensis* |  | 0.021% | 99.76% | KP697285.1 |
| OTU2672 | Brachycera | Drosophilidae | *Leucophenga spinifera* |  | 0.023% | 100.00% | MG784285.1 |
| OTU2885 | Brachycera | Drosophilidae | *Liodrosophila aerea* |  | 0.483% | 98.80% | JF273080.1 |
| OTU5761 | Brachycera | Drosophilidae | *Mycodrosophila erecta* | yes | 0.031% | 100.00% | LC194108.1 |
| OTU820 | Brachycera | Drosophilidae | *Mycodrosophila gratiosa* | yes | 0.095% | 99.76% | LC194119.1 |
| OTU4312 | Brachycera | Drosophilidae | *Mycodrosophila poecilogastra* | yes | 0.020% | 98.80% | LC194024.1 |
| OTU4806 | Brachycera | Drosophilidae | *Paramycodrosophila nakamurai* | yes | 0.072% | 98.33% | LC194096.1 |
| OTU5774 | Brachycera | Drosophilidae | *Phortica chi* | yes | 0.021% | 100.00% | MN228854.1 |
| OTU1551 | Brachycera | Drosophilidae | *Phortica flexuosa* | yes | 0.008% | 100.00% | MN228877.1 |
| OTU166 | Brachycera | Drosophilidae | *Phortica gamma* | yes | 0.053% | 99.76% | KJ130790.1 |
| OTU53 | Brachycera | Drosophilidae | *Phortica pseudotau* | yes | 0.004% | 99.28% | KJ130850.1 |
| OTU6476 | Brachycera | Drosophilidae | *Phortica speculum* | yes | 0.004% | 100.00% | KJ083036.1 |
| OTU825 | Brachycera | Drosophilidae | *Phortica tau* | yes | 0.257% | 99.52% | MN228909.1 |
| OTU4444 | Brachycera | Drosophilidae | *Scaptodrosophila coracina* | yes | 0.029% | 99.03% | MF069143.1 |
| OTU5832 | Brachycera | Drosophilidae | *Scaptomyza pallida* | yes | 0.037% | 99.52% | KY839828.1 |
| OTU4447 | Brachycera | Drosophilidae | *Stegana kanmiyai* | yes | 0.031% | 99.76% | MH373092.1 |
| OTU6874 | Brachycera | Dryomyzidae | *Dryomyza formosa* | yes | 0.025% | 98.56% | KC427131.1 |
| OTU6766 | Brachycera | Empididae | *Heleodromia immaculata* |  | 0.001% | 99.76% | KT225295.1 |
| OTU4131 | Brachycera | Empididae | *Ocydromia glabricula* | yes | 0.004% | 96.65% | MG823423.1 |
| OTU5495 | Brachycera | Ephydridae | *Hydrellia nobilis* | yes | 0.003% | 96.65% | HM412473.1 |
| OTU6690 | Brachycera | Lauxaniidae | *Cestrotus liui* | yes | 0.002% | 95.69% | KX372559.1 |
| OTU5707 | Brachycera | Lauxaniidae | *Homoneura mayrhoferi* | yes | 0.032% | 98.80% | KR262638.1 |
| OTU3995 | Brachycera | Muscidae | *Coenosia atra* |  | 0.529% | 95.22% | MN868789.1 |
| OTU1544 | Brachycera | Muscidae | *Dichaetomyia bibax* |  | 0.158% | 100.00% | KP161685.1 |
| OTU4939 | Brachycera | Muscidae | *Dichaetomyia bibax* |  | 0.010% | 98.56% | KY842841.1 |
| OTU3493 | Brachycera | Muscidae | *Limnophora triangula* |  | 0.013% | 97.37% | KC626326.1 |
| OTU5039 | Brachycera | Muscidae | *Lispocephala miki* |  | 0.024% | 99.75% | KP161692.1 |
| OTU3816 | Brachycera | Muscidae | *Morellia simplex* |  | 0.010% | 95.69% | MT410788.1 |
| OTU3464 | Brachycera | Muscidae | *Muscina pascuorum* |  | 0.003% | 99.76% | JX861445.1 |
| OTU5579 | Brachycera | Phoridae | *Megaselia hilaris* | yes | 0.006% | 97.37% | KP693295.1 |
| OTU2541 | Brachycera | Phoridae | *Megaselia rufipes* |  | 0.006% | 98.80% | MN520932.1 |
| OTU1318 | Brachycera | Phoridae | *Megaselia variana* | yes | 0.000% | 97.38% | KX774935.1 |
| OTU448 | Brachycera | Pipunculidae | *Tomosvaryella palliditarsis* | yes | 0.033% | 99.28% | MT410821.1 |
| OTU308 | Brachycera | Sarcophagidae | *Phallosphaera gravelyi* |  | 0.065% | 100.00% | KF038011.1 |
| OTU3962 | Brachycera | Sarcophagidae | *Sarcophaga antilope* |  | 0.012% | 100.00% | NC_039827.1 |
| OTU367 | Brachycera | Sarcophagidae | *Sarcophaga genuforceps* |  | 0.002% | 99.52% | MW592364.1 |
| OTU6806 | Brachycera | Scathophagidae | *Scathophaga furcata* | yes | 0.005% | 95.69% | KT107653.1 |
| OTU6222 | Brachycera | Sphaeroceridae | *Coproica hirtula* |  | 0.001% | 98.56% | JF873119.1 |
| OTU5886 | Brachycera | Sphaeroceridae | *Crumomyia annulus* | yes | 0.006% | 97.13% | JF875209.1 |
| OTU6035 | Brachycera | Sphaeroceridae | *Spelobia bifrons* | yes | 0.012% | 99.52% | KR666736.1 |
| OTU6507 | Brachycera | Stratiomyidae | *Ptecticus aurifer* |  | 0.024% | 99.28% | MN604259.1 |
| OTU3777 | Brachycera | Syrphidae | *Allograpta medanensis* | yes | 0.003% | 99.52% | MT449481.1 |
| OTU5471 | Brachycera | Syrphidae | *Episyrphus balteatus* |  | 0.782% | 100.00% | MN621939.1 |
| OTU4027 | Brachycera | Syrphidae | *Eristalis cerealis* |  | 0.081% | 100.00% | KT175588.1 |
| OTU2250 | Brachycera | Syrphidae | *Eumerus flavitarsis* | yes | 0.036% | 97.61% | MN621962.1 |
| OTU5936 | Brachycera | Syrphidae | *Fagisyrphus cinctus* | yes | 0.068% | 96.89% | KM270869.1 |
| OTU6425 | Brachycera | Syrphidae | *Ferdinandea luteola* | yes | 0.018% | 100.00% | MH521928.1 |
| OTU468 | Brachycera | Syrphidae | *Grzegorzekia hungarica* | yes | 0.035% | 95.93% | MH114424.1 |
| OTU6590 | Brachycera | Syrphidae | *Helophilus lapponicus* | yes | 0.008% | 97.44% | MN682305.1 |
| OTU4103 | Brachycera | Syrphidae | *Helophilus virgatus* |  | 1.134% | 100.00% | MN148445.1 |
| OTU5847 | Brachycera | Syrphidae | *Meliscaeva cinctella* |  | 0.427% | 95.69% | GU689993.1 |
| OTU6253 | Brachycera | Syrphidae | *Microdon stilboides* |  | 0.206% | 95.96% | HF569342.1 |
| OTU5169 | Brachycera | Tachinidae | *Compsilura concinnata* |  | 0.013% | 100.00% | LC516549.1 |
| OTU6784 | Brachycera | Tachinidae | *Exorista patelliforceps* | yes | 0.018% | 98.13% | AB700026.1 |
| OTU6446 | Brachycera | Tachinidae | *Linnaemya rossica* | yes | 0.027% | 97.13% | KX843890.1 |
| OTU6554 | Brachycera | Tachinidae | *Medina luctuosa* |  | 0.008% | 96.65% | HQ581783.1 |
| OTU3598 | Brachycera | Tachinidae | *Metadrinomyia flavifrons* | yes | 0.008% | 95.45% | KR262646.1 |
| OTU2392 | Brachycera | Tachinidae | *Mycodrosophila basalis* | yes | 0.005% | 100.00% | LC193982.1 |
| OTU2023 | Brachycera | Tachinidae | *Peribaea tibialis* |  | 0.018% | 98.80% | KX843900.1 |
| OTU4112 | Brachycera | Tachinidae | *Phorocerosoma vicarium* |  | 0.001% | 98.13% | AB700038.1 |
| OTU1545 | Brachycera | Tachinidae | *Senometopia intermedia* | yes | 0.019% | 97.13% | KX844385.1 |
| OTU1642 | Brachycera | Tachinidae | *Senometopia intermedia* | yes | 0.017% | 95.91% | KX844385.1 |
| OTU6511 | Brachycera | Tachinidae | *Senometopia intermedia* | yes | 0.117% | 95.22% | KX844385.1 |
| OTU3454 | Brachycera | Tachinidae | *Siphona intrudens* | yes | 0.005% | 97.13% | KT099362.1 |
| OTU6436 | Brachycera | Tachinidae | *Siphona paludosa* |  | 0.001% | 100.00% | KX843873.1 |
| OTU5695 | Brachycera | Tachinidae | *Siphona subarctica* | yes | 0.063% | 96.89% | KX843737.1 |
| OTU3643 | Brachycera | Tachinidae | *Thelaira nigripes* |  | 0.004% | 96.89% | KX844290.1 |
| OTU2720 | Brachycera | Tachinidae | *Vibrissina turrita* |  | 0.155% | 97.85% | KX843925.1 |
| OTU4036 | Brachycera | Tachinidae | *Zenillia libatrix* |  | 0.064% | 95.69% | KX843781.1 |
| OTU3528 | Brachycera | Tephritidae | *Dacus trimacula* | yes | 0.002% | 99.76% | MT318718.1 |
| OTU5167 | Brachycera | Tephritidae | *Trupanea radifera* | yes | 0.012% | 96.40% | KM634864.1 |
| OTU1599 | Brachycera | Xylophagidae | *Metylophorus wuyinicus* | yes | 0.002% | 98.66% | MT408503.1 |
| **173 OTUs** | **5 higher taxa** | **31 families** | **163 species identified** | **89 species**  **new to China** | **15.4% of total abundance** |  |  |

**Table S6.** Pairwise comparison of the effects of elevation and seasonality on dipteran community composition by PERMANOVA testing.

| **Groups** | **Df** | **Sums of**  **squares** | **Mean**  **squares** | **F.Model** | **Variation**  **(R^2^)** | ***P*** |
| --- | --- | --- | --- | --- | --- | --- |
| Elevation | | | | | | |
| A vs B | 1 | 1.239 | 1.239 | 5.548 | 0.182 | 0.001 |
| A vs C | 1 | 1.451 | 1.451 | 5.438 | 0.179 | 0.001 |
| A vs D | 1 | 1.859 | 1.859 | 7.676 | 0.235 | 0.001 |
| A vs E | 1 | 1.962 | 1.962 | 8.558 | 0.255 | 0.001 |
| B vs C | 1 | 0.671 | 0.671 | 2.411 | 0.066 | 0.014 |
| B vs D | 1 | 0.892 | 0.892 | 3.427 | 0.092 | 0.001 |
| B vs E | 1 | 1.183 | 1.183 | 4.718 | 0.122 | 0.001 |
| C vs D | 1 | 0.823 | 0.823 | 2.817 | 0.077 | 0.003 |
| C vs E | 1 | 0.999 | 0.999 | 3.535 | 0.094 | 0.001 |
| D vs E | 1 | 0.470 | 0.470 | 1.775 | 0.050 | 0.067 |
| Seasonality | | | | | | |
| MayJ vs JulA | 1 | 2.093 | 2.093 | 8.563 | 0.141 | 0.001 |
| MayJ vs SepO | 1 | 3.174 | 3.174 | 12.622 | 0.195 | 0.001 |
| JulA vs SepO | 1 | 2.470 | 2.470 | 10.334 | 0.166 | 0.001 |

Note: Elevations: A, 320–500 m; B, 500–700 m; C, 700–850 m; D, 850–1000 m; E, 1000–1150 m. Month abbreviations: MayJ, May to June; JulA, July to August; SepO, September to October.


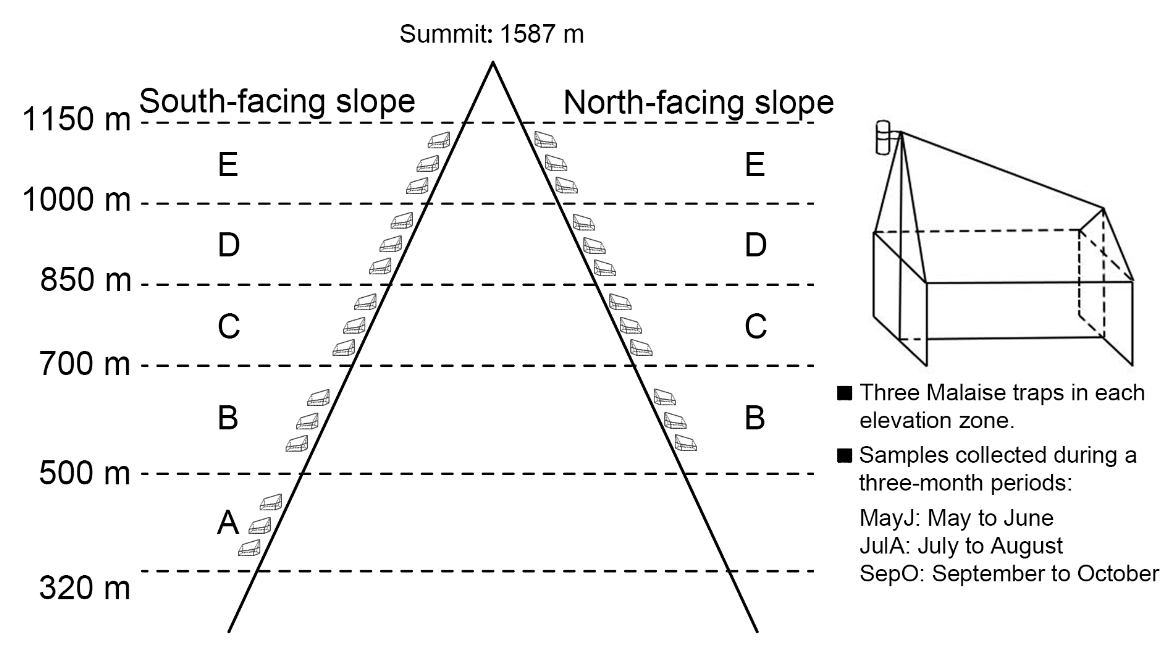


**Figure S1.** Diagram of Malaise trap distribution on Tianmu Mountain in this study.


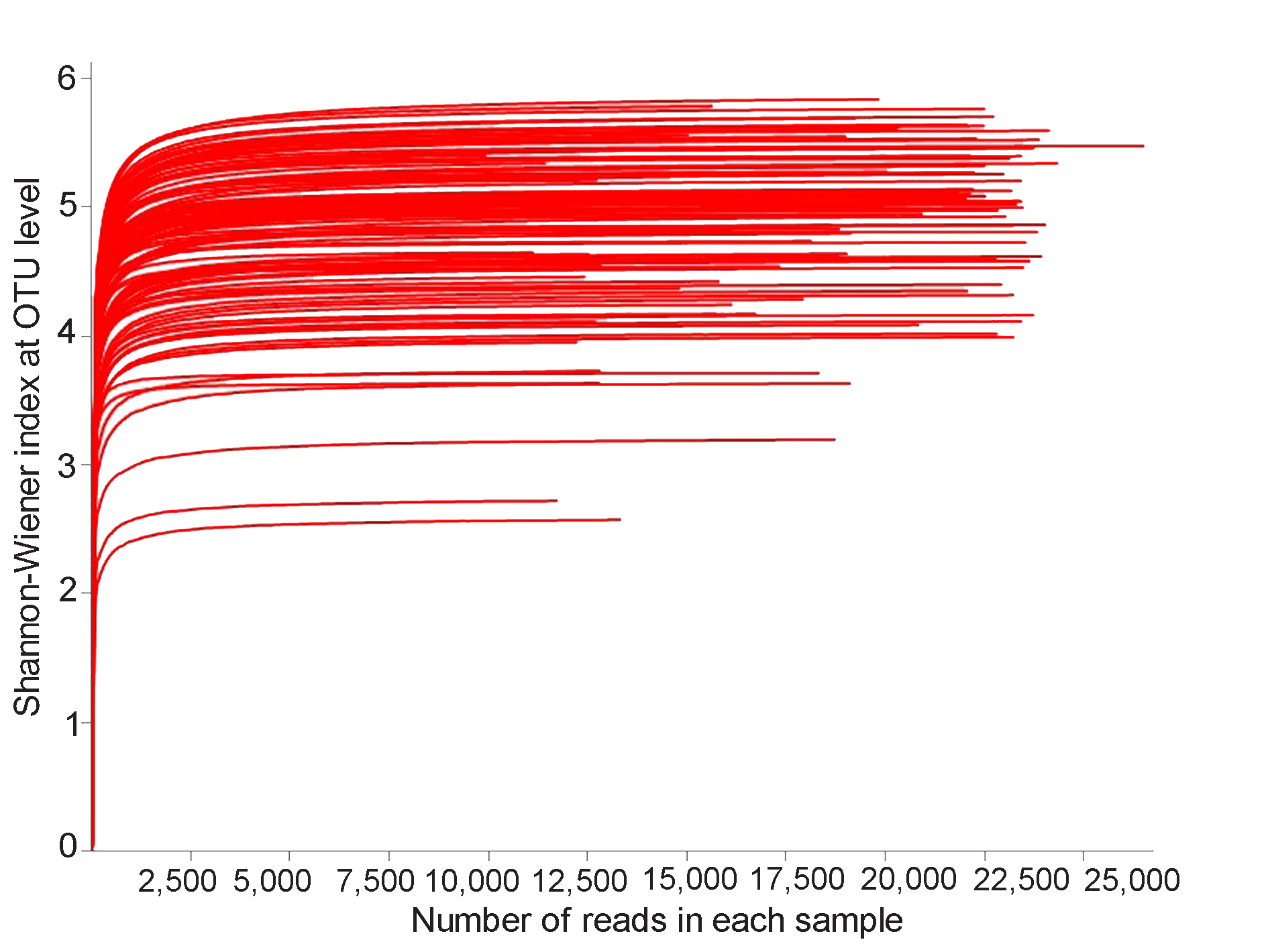


**Figure S2.** The rarefaction curves of Shannon-Wiener index of OTUs clustered at 95% similarity for all the sequenced dipteran samples.


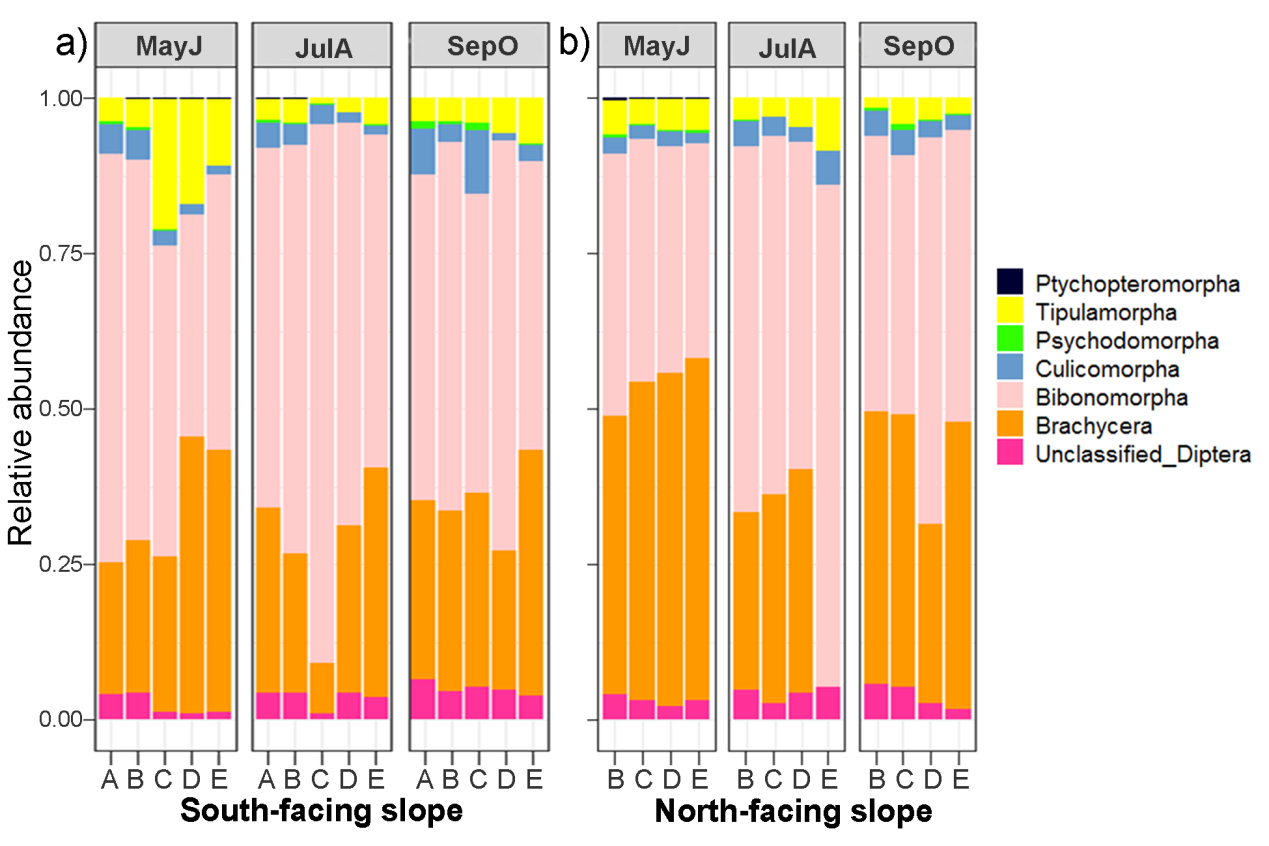


**Figure S3.** Diptera fauna abundance at higher level across different periods and elevations. Peroid abbreviations: MayJ, May to June; JulA, July to August; SepO, September to October. Elevation zones: A, 320‒500 m; B, 500‒700 m; C, 700–850 m; D, 850‒1,000 m; E, 1,000‒1,150 m.


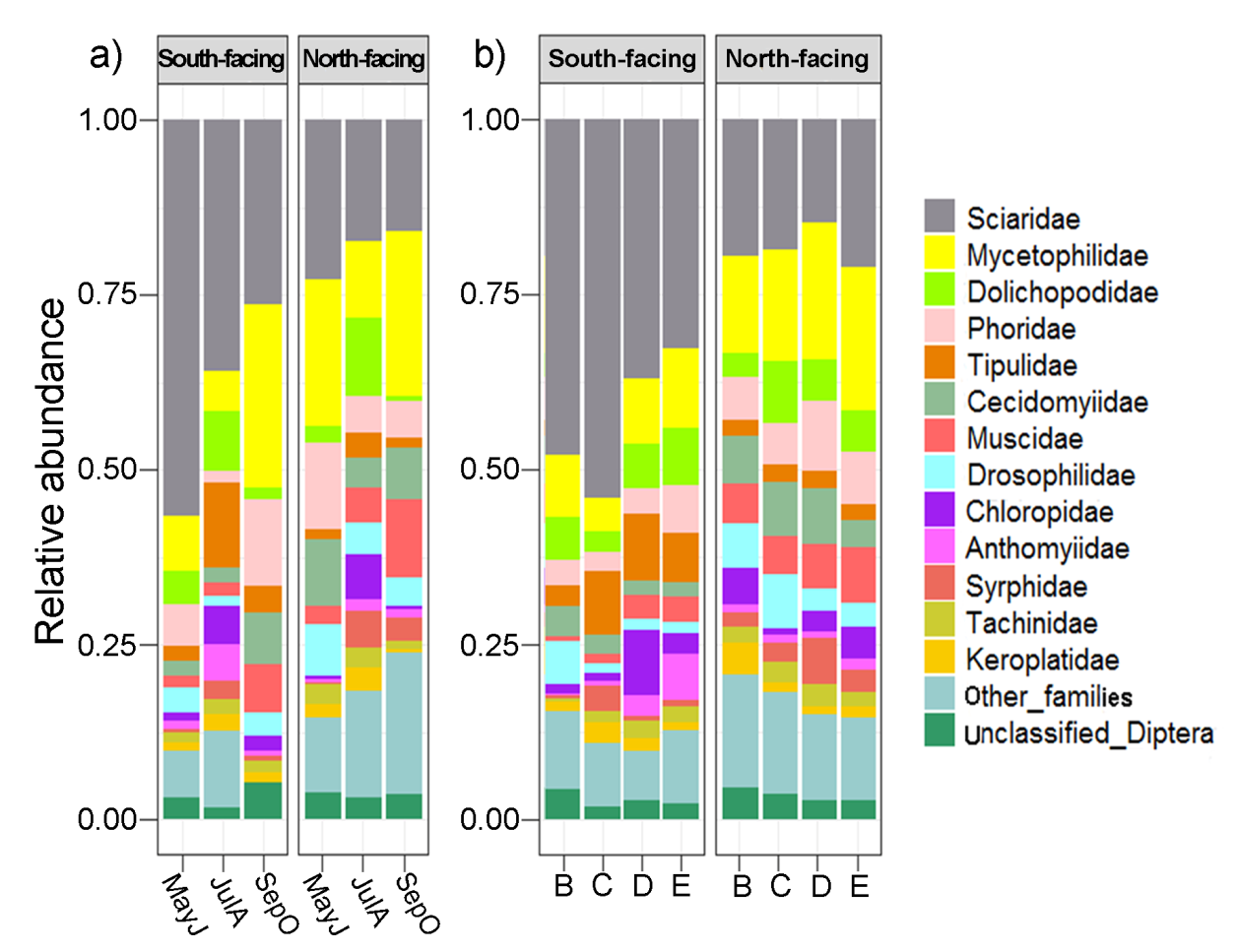


**Figure S4.** Diptera abundance at family level for the south-facing and north-facing slopes. Data shown are the 15 most species rich families and the rest are assigned to the "other_families". Families are represented by blocks in different color. Period abbreviations: MayJ, May to June; JulA, July to August; SepO, September to October. Elevation zones: A, 320‒500 m; B, 500‒700 m; C, 700–850 m; D, 850‒1,000 m; E, 1,000‒1,150 m.


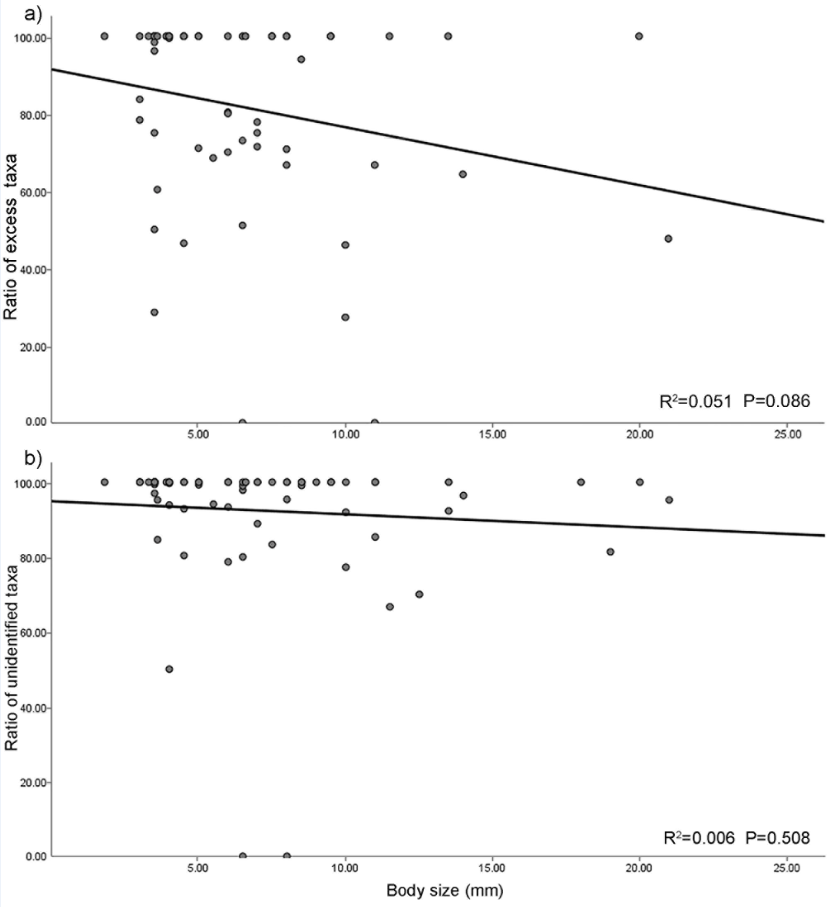


**Figure S5.** The relationships between the ratio of unknown taxa and median body size (mm) among different families.


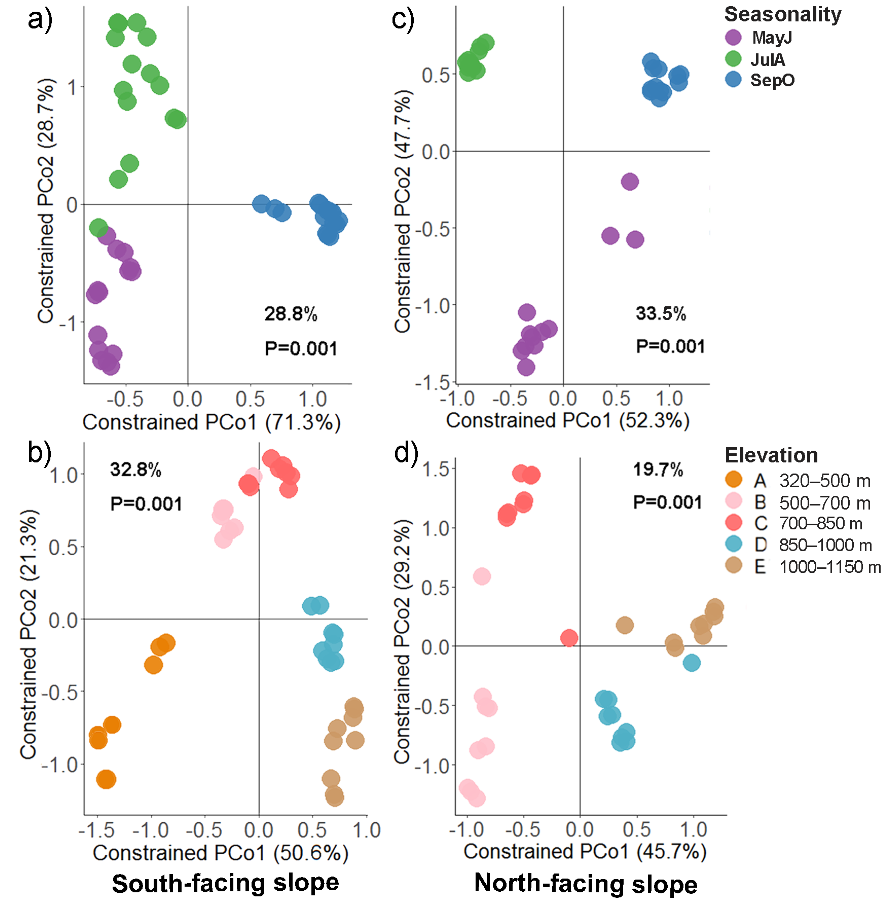


**Figure S6.** Constrained effects of seasonality and elevation on dipteran diversity, by partial canonical analysis of principal coordinates using the Bray-Curtis distances. (a, c) Constrained to the month periods and conditioned by the elevations. (b, d) Constrained to the elevations and conditioned by the month periods.


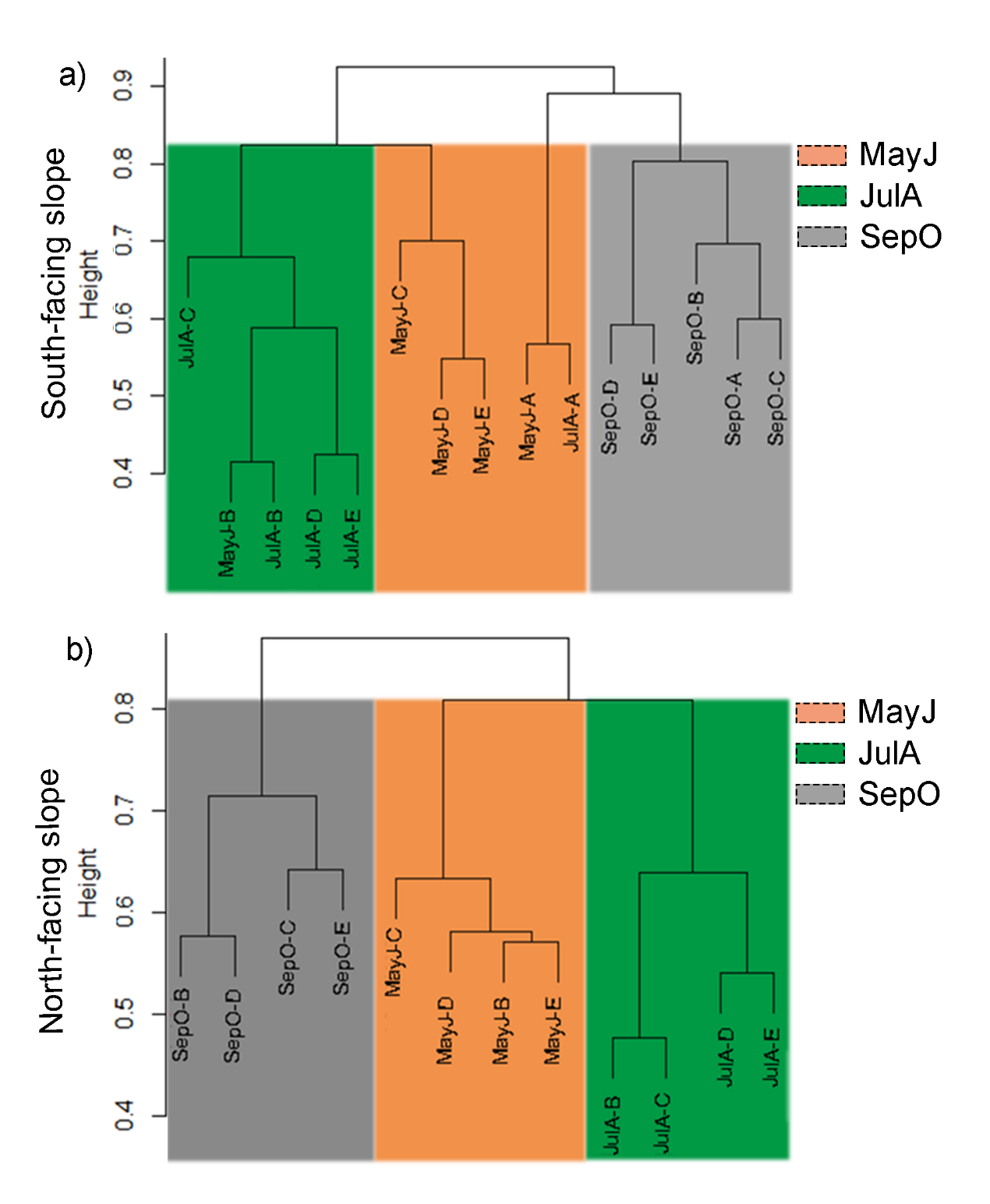


**Figure S7.** The Cluster dendrogram (Bray-Curtis algorithm) of community composition at OTU level for the south-facing and north-facing slopes. Period abbreviations: MayJ: May to June; JulA: July to August; SepO: September to October. Elevation zones: A, 320‒500 m; B, 500‒700 m; C, 700–850 m; D, 850‒1,000 m; E, 1,000‒1,150 m.
